# Bone marrow immune cells and drug resistance in acute myeloid leukemia

**DOI:** 10.3389/ebm.2025.10235

**Published:** 2025-02-11

**Authors:** Miao Zhang, You Yang, Jing Liu, Ling Guo, Qulian Guo, Wenjun Liu

**Affiliations:** ^1^ Department of Pediatrics (Hematological Oncology), Children Hematological Oncology and Birth Defects Laboratory, The Affiliated Hospital of Southwest Medical University, Luzhou, Sichuan, China; ^2^ Sichuan Clinical Research Center for Birth Defects, The Affiliated Hospital of Southwest Medical University, Luzhou, Sichuan, China

**Keywords:** leukemia, immune cells, drug resistance, bone marrow, immunosuppressive microenvironment

## Abstract

In recent years, the relationship between the immunosuppressive niche of the bone marrow and therapy resistance in acute myeloid leukemia (AML) has become a research focus. The abnormal number and function of immunosuppressive cells, including regulatory T cells (Tregs) and myeloid-derived suppressor cells (MDSCs), along with the dysfunction and exhaustion of immunological effector cells, including cytotoxic T lymphocytes (CTLs), dendritic cells (DCs) and natural killer cells (NKs), can induce immune escape of leukemia cells and are closely linked to therapy resistance in leukemia. This article reviews the research progress on the relationship between immune cells in the marrow microenvironment and chemoresistance in AML, aiming to provide new ideas for the immunotherapy of AML.

## Impact statement

Over the past few years, tolerance to chemical therapy in leukemia has become a significant challenge in treatment. The development of the leukemia immune niche significantly contributes to resistance in leukemia. Our article discusses the key roles of several immune cells in the immune microenvironment of AML in the development of resistance. Abnormalities in the number and function of immune cells in the AML immune microenvironment are vital in leukemia resistance. Therefore, immunotherapy is an important strategy to combat acute myeloid leukemia resistance and improve patient prognosis.

## Introduction

AML represents the most prevalent form of leukemia in adults and ranks as the second most frequently diagnosed leukemia in children. Currently, under conventional intensive chemotherapy, the complete remission (CR) rate ranges from 60% to 85% in adults younger than 60 years, while it decreases to 40%–60% in elderly patients aged 60 years or older [1]. Nevertheless, the overall survival (OS) rate over 5 years is only approximately 27% among patients with AML [2]. Although the CR rate of children with AML is high at approximately 90% compared with that of adults, the 3-year event-free survival (EFS) rate remains suboptimal at 45%, with the OS rate also being only at 65%, and nearly half of the children are resistant to relapse [3]. Therefore, relapse with drug resistance continues to be a significant factor contributing to poor prognosis among AML patients. The development for drug resistance in AML patients may be associated with leukemia cells evading immune responses and the progression of the minimal residual disease (MRD) in the bone marrow (BM) [4]. With the continuous proliferation of tumor cells, changes in immunogenicity, the recruitment of inhibitory immune cells, and the dysfunction and depletion of effector immune cells induce the immune evasion of leukemia cells. Among them, the strengthening of inhibitory immune cells and the weakening of effector immune cells could serve as a key contributor to the development of tumor cell resistance. Our review summarizes the role of immune cells in the bone marrow microenvironment of drug-resistant AML, helping to identify new therapeutic targets, optimize chemotherapy regimens, and improve the prognosis for AML patients.

## Tregs and drug resistance in AML

Tregs, a specialized subset of T cells with immunosuppressive properties, are essential for preserving immune tolerance and facilitating the immune evasion of tumor cells. Tregs can be divided into two categories based on their origin and function: natural Tregs (nTregs) and induced Tregs (iTregs). nTregs, characterized by the markers CD4^+^CD25+Foxp3+, originate and mature in the thymus and possess intrinsic immunosuppressive functions, primarily maintaining self-tolerance and immune homeostasis by regulating effector immune responses. Unlike nTregs, iTregs are induced from CD4^+^ T cells through external signals in peripheral blood (PB). Based on cellular phenotype and function, iTregs are divided into Foxp3+ Treg, T helper 3 cell (Th3), and Type 1 regulatory T cell (Tr1). According to their immunophenotypes, Tregs can be categorized into three types, including CD4^+^ Tregs, CD8^+^ Tregs and double-negative Tregs (DN Tregs, CD4^−^CD8^−^ Tregs). There were slight differences in the functions of Tregs with different phenotypes and their specific roles in AML, as shown in Table 1. Additionally, DN Tregs possess unique immunoregulatory capabilities that can induce the functional inactivation of effector T lymphocytes (Teffs) while also suppressing the immune response of NKs via the secretion of perforin [11]. McIver et al. suggested that DN Tregs are essential for immune tolerance after allogeneic hematopoietic stem cell transplantation (allo-HSCT) by regulating the diversity of the TCR repertoire and suppressing the excessive proliferation of immune-reactive T cells, which is especially critical for preventing graft-versus-host disease (GVHD) [12]. Ford et al. revealed that LPS-activated allogeneic antigen-presenting Cells (APCs) can promote the expansion of DN Tregs, which in turn enhances their immune-regulatory function by killing B cells through a perforin-dependent pathway [13]. Studies have shown that re-infusion of human DN Treg cells, after *ex vivo* expansion, can effectively inhibits the growth of autologous T and B lymphocytes and alleviate GVHD [14]. Additionally, pretreatment with rapamycin (an mTOR inhibitor) further enhances their immunoregulatory function, highlighting the potential clinical application of DN Treg cells in therapeutic settings [14]. However, the role of DN Tregs in AML is still in the early stages, requiring further investigation.

**TABLE 1:** Phenotype and function of Tregs.

Type		Origin	Immunophenotype	Main function	Role in AML
CD4+Tregs	Natural Tregs (nTregs)	Thymus	CD4+, CD25+, Foxp3+ Tregs	Suppress autoreactive T cells through contact-dependent mechanisms and inhibitory cytokines to maintain self-tolerance	Promote immune escape by inhibiting CD8+ T cells, DCs and NKs. High levels in AML correlate with poor prognosis.
	Induced Tregs (iTregs)	Peripheral tissues	Tr1 (CD4+, IL-10high)	Secrete elevated amounts of IL-10 and TGF-β, with minimal Foxp3 expression, and release granzyme B and perforin to mediate cytotoxicity [5]	Regulate the tumor microenvironment through IL-10 and TGF-β, induce the increase of Tregs, and indirectly promote tumor immune escape while suppressing the immune response
			Th3 (CD4+, TGF-βhigh)	Primarily secrete TGF-β, induce the differentiation of CD4+ T cells into Tregs, and upregulate Foxp3 expression [6]	
			CD4+, Foxp3+ Tregs	Differentiated from CD4+T cells under the induction of TGF-β and IL-2, inhibit effector T-cell function, maintain peripheral tolerance, and inhibit autoimmune responses [7]	
CD8+ Tregs		Thymus or Peripheral tissues	CD8+ T cells, partially expressing Foxp3	Secrete IL-10 and TGF-β to inhibits the activity of CD4+ T cells and B cells, reduce the release of inflammatory cytokines through the CTLA-4 and PD-1 pathways, directly induce target cells death via the Fas/Fasl and perforin/granzyme B pathways,thereby enhancing the anti-tumor effect [8]	CD8+ Tregs exert their unique therapeutic potential and advantages by secreting immunosuppressive factors and specifically regulating immune responses, thereby alleviating GVHD while maintaining the GVL effect. However, research on CD8+ Tregs in AML is limited
CD4-CD8- Tregs		Thymus and peripheral tissues [9]	Double negative T cells,CD4-, CD8-	Express IFN-γ, TNF-α, Ly6A, FcRγ, and CXCR5; acquire MHC-peptide complexes from antigen-presenting cells; and exert immunosuppression through Fas/Fas ligand interactions [10]	mechanisms include suppression of effector cells and promotion of a tolerogenic microenvironment

### Cytokines secreted by Tregs are involved in drug resistance in AML

**FIGURE 1:**
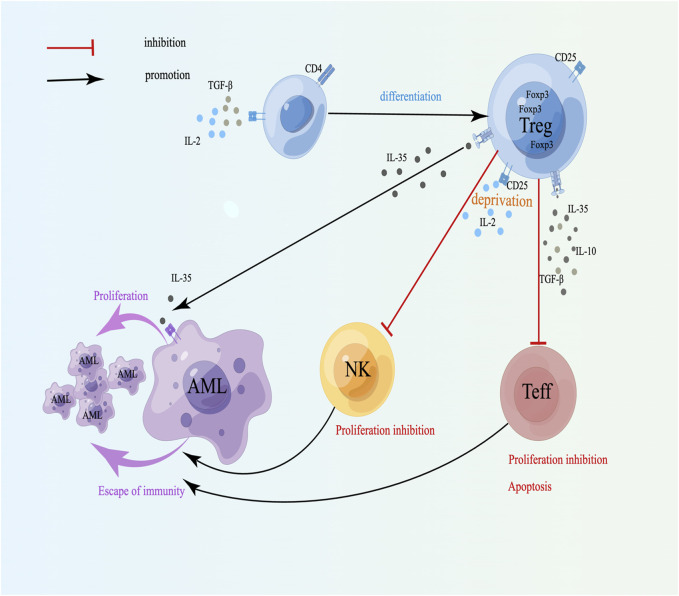
Cytokines secreted by Tregs and drug resistance in AML. TGF-β can cooperate with IL-2 to induce naive T lymphocytes to differentiate into Tregs; CD25^+^ Tregs inhibit NK and Teff proliferation and induce apoptosis through competitive binding to IL-2; IL-35 can directly promote the proliferation of leukemia cells. A schematic diagram is created by Figdraw (www.figdraw.com).

A high frequency of CD4^+^CD25+Foxp3+ Tregs is closely associated with immune tolerance and chemoresistance relapse in AML. The transcription factor Foxp3, when highly expressed in the nucleus, plays a vital role in triggering suppressive activity of Tregs and stabilizes of their phenotype and functional properties. [15], and its mechanism may be related to DNA methyltransferases at the Foxp3 locus [16]. Foxp3+ Tregs maintain immune tolerance by suppressing immune responses through the secretion of inhibitory cytokines, including IL-10, TGF-β, and IL-35, as well as the expression of immune regulatory molecules [17, 18]. These molecules interact with receptors on immune cells to exert potent immunosuppressive effects, making Tregs key factors in facilitating immune evasion of tumors. Studies have shown that the elevated levels of IL-35 are linked to unfavorable outcomes in AML [19, 20]. TGF-β can cooperate with IL-2 to upregulate the production of Foxp3 and promote the transformation of naive CD4^+^ T lymphocytes to Tregs [21] (Figure 1). IL-35 plays a dual role by suppressing the proliferation of CD4^+^CD25^−^ effector T cells while simultaneously promoting the growth and preventing the apoptosis of AML cells [22] (Figure 1). Compared with healthy individuals, AML patients present significantly elevated levels of Tregs in the PB and BM, which are positively correlated with IL-35 expression [18, 23]. Conversely, the proportions of cytotoxic lymphocytes and B cells are relatively low [24]. It is closely related to recurrence and drug resistance in AML. Elevated IL-10 levels and reduced IL-6 levels are linked to OS rates in AML patients and could act as potential biomarkers for predicting disease progression in AML patients [25]. Furthermore, studies have shown that a high-frequency single-nucleotide polymorphism (SNP) at position −819 of IL-10 has been identified as a factor that raises the risk of AML [26]. In AML patients, high expression of TGF-β1 may inhibit the immune function of NKs by phosphorylating SMAD and downregulating Natural Killer Group 2 Member D Receptor (NKG2DR) expression [27]. In addition, studies have demonstrated that TGF-β1 can suppress the antitumor immunity of NK cells through enhancing the SMAD3 signaling to induce CD96 expression on NK cells, thereby reducing IFN-γ production [28]. Importantly, transcriptomic analysis of HL60 cells (M3 subtype) revealed that TGF-β/activin signaling represents a promising target to overcome drug resistance in AML [29]. Studies indicate that relapse after allogeneic hematopoietic stem cell transplantation (allo-HSCT) may be linked to increased levels of TGF-β1, which strongly suppress mTORC1 activity, mitochondrial oxidative phosphorylation, cell proliferation, and cytotoxic functions of NKs in the BM, leading to their functional impairment [30]. In addition, Tregs promote the polarization of macrophages into M2-type tumor-associated macrophages (M2-TAMs) by secreting IL-10 and TGF-β. M2-TAMs exhibit tumor-promoting functions, such as degrading the tumor extracellular matrix, promoting angiogenesis, and recruiting immunosuppressive cells, thereby facilitating tumor progression and distant metastasis [31]. Research has demonstrated that AML blasts recruit M2-TAMs and Tregs, resulting in their high infiltration into the BM, which is linked to poor prognosis [32]. To conclude, the inhibitory cytokines secreted by Tregs can enhance immune tolerance and immune escape by directly inhibiting the function of effector immune cells, promoting differentiation and function of Tregs, and regulating immune microenvironment.

Moreover, CD4^+^CD25^+^ Tregs possess high-affinity IL-2 receptor (CD25), which depletes IL-2, thereby suppressing the proliferation of Teff cells and promoting their apoptosis (Figure 1). IL-2 can stimulate the growth of NKs, however, when Tregs and NKs are cocultured, Tregs can inhibit the proliferation of NKs by competing with IL-2 [33, 34], indicating that the affinity between Tregs and IL-2 may be dominant, which can weaken the immune ability and facilitate the escape of tumor cells (Figure 1). CD8+Foxp3- Tregs can inhibit the function of Teffs through intercellular contact and the release of suppressive cytokines like IL-10 and TGF-β [35]. Therefore, Tregs mainly downregulate the quantity and activity of Teffs by secreting different soluble negative immune molecules and suppress the growth of Teffs and NKs by competing with and depleting IL-2, which can cause immune escape of tumor cells and induce drug resistance in AML.

### Surface molecules of Tregs and drug resistance in AML

**FIGURE 2:**
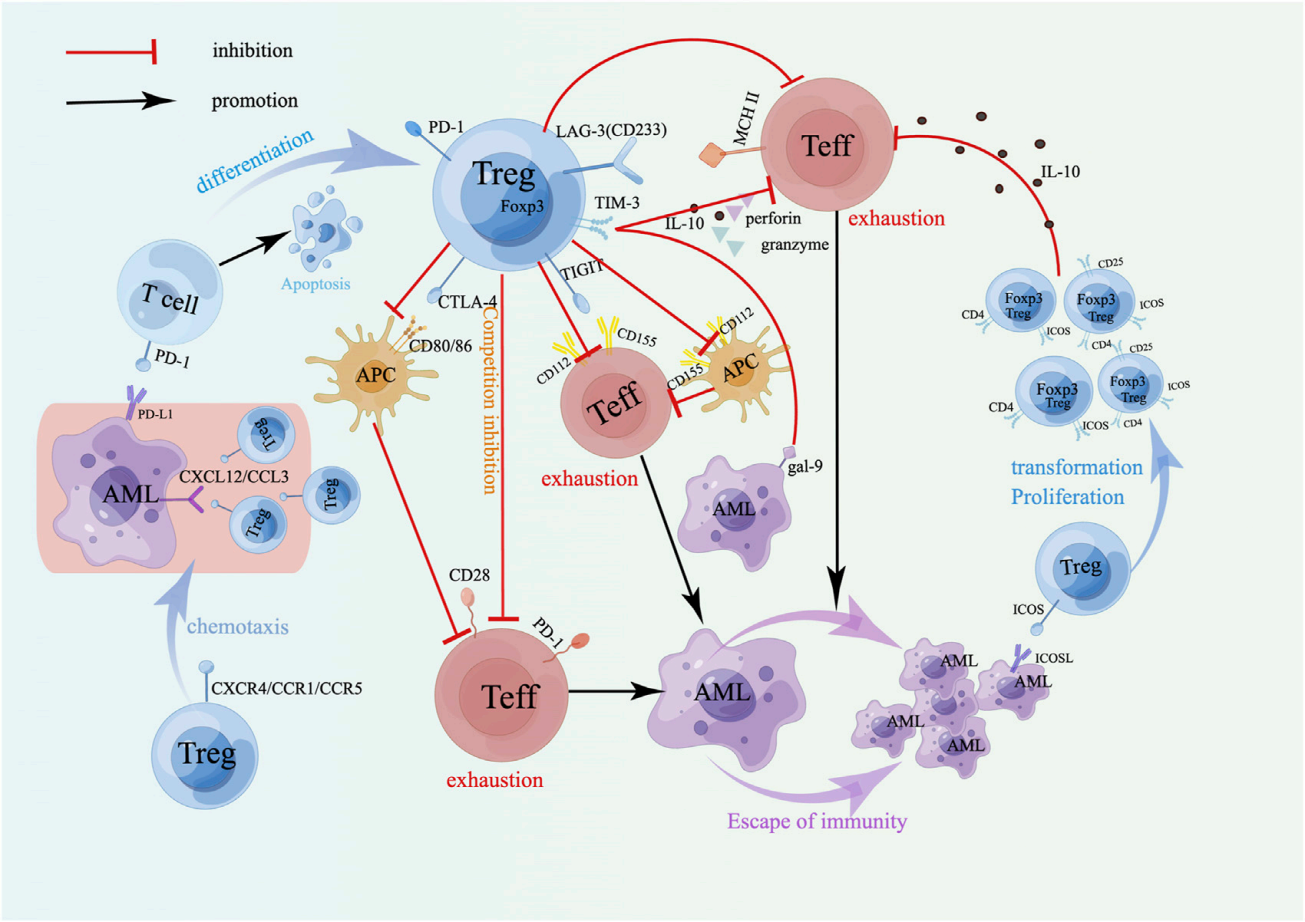
Surface molecules of Tregs and drug resistance in AML. The coinhibitory receptor on Tregs mainly induces immune escape of AML cells by inhibiting APCs and depleting Teffs. The combination of the costimulatory receptor ICOS on Tregs and ICOSL on AML cells can induce the transformation and proliferation of Tregs, produce more IL-10, and play a stronger immunosuppressive role. Treg-expressed chemokine receptors promote their accumulation at the tumor site to exert an immunosuppressive effect. A schematic diagram is created by Figdraw (www.figdraw.com).

A variety of coinhibitory receptors (CIRs), including cytotoxic T lymphocyte-associated protein 4 (CTLA-4), T-cell immunoglobulin and mucin domain 3 (TIM-3), programmed cell death protein 1 (PD-1), lymphocyte activation 3 (LAG-3, CD223) and T-cell immunoglobulin and immunoreceptor tyrosine-based inhibitory motif domains (TIGIT), expressed on the surface of Tregs are critical for their immunosuppressive activity. Studies have shown that the highly expressed coinhibitory molecule CTLA-4 on Tregs competes with CD28 on Teff cells to attach to CD80/86 on the surface of APCs, resulting in the inhibition of Teffs and APCs activation and increased apoptosis, thereby weakening the immune killing effect of Teffs on tumor cells [36]. Compared with those of healthy controls, AML patients exhibit significantly higher levels of CTLA-4 and LAG-3, which is closely related to poor prognosis [37, 38]. Interestingly, the CTLA-4 expression level in individuals with APL is markedly higher compared to those with non-M3 subtypes [39]. Research conducted by Davids demonstrated that CTLA-4-specific antibody ipilimumab in treating AML patients who relapsed following allo-HSCT can promote the infiltration of cytotoxic CD8^+^ T lymphocytes and the expansion of Teff subsets, suggesting that ipilimumab is feasible for managing drug-resistant relapsed patients [40]. Studies have shown that the combination of PD-L1 highly expressed on AML cells and PD-1 on the surface of T lymphocytes can induce them to differentiate and expand into Tregs that highly express Foxp3 and PD-1, and these Tregs release suppressive cytokines, including IL-10 and IL-35, enabling tumor cell immune escape [41]. Moreover, PD-1 on T lymphocytes interacts with PD-L1 on tumor cells, which can also induce T lymphocyte apoptosis [42]. Chen et al. analyzed The Cancer Genome Atlas (TCGA) database and verified 62 AML bone marrow samples and indicated that the combined high expression of PD1, PD-L1, PD-L2 and CTLA-4 was linked to reduce OS rates [43]. In patients with a high AML cell burden, the bone marrow shows a significant increase in PD-1+ Tregs and PD-1+ TIGIT+ Tregs. Interestingly, the elevated PD-1 is closely linked to lactate secretion by AML blasts [44]. TIGIT is expressed only on lymphocytes, especially nTregs, which can promote the differentiation of Tregs and combine with CD112 and CD155 [45]. TIGIT+ Tregs mainly inhibit the differentiation and proliferation of Th1 cells and Th17 cells via a mechanism dependent on fibrinogen-like protein 2 (FGL2) [45]. Thus, TIGIT may be an important immunosuppressive molecule related to the immunosuppressive function of Tregs, which are involved in tumor immune escape and resistance. Relapse, the primary cause of mortality in AML patients following allo-HSCT, is strongly linked to elevated TIGIT expression on CD4^+^ T cells [44]. LAG-3 shares high homology with CD4 [46], is highly expressed on Tregs [47], and has a high affinity for major histocompatibility complex class II (MHC II). The combination of LAG-3 and MHC II on CD4^+^ T cells induces an inhibitory pathway mediated by the activation of immune body tyrosine kinase [48], inhibits the immunity of T cells so that targeting the LAG-3/MHC II signaling pathway helps to promote the immune effect of CD4^+^ T cells against tumors, indicating that LAG-3 is critical for Treg-mediated immunosuppression and may serve as a novel therapeutic strategy in AML. Research has indicated that LAG3 not only can bind to MHC II [49] but can also interact with soluble liver-secreted fibrinogen-like protein 1 (FGL-1), thereby inhibiting both autoimmune and antitumor immunity [50]. Additionally, Tregs in AML patients express high levels of LAG3, which can reduce the activation of CD8^+^ T cells and is linked to poor outcomes [51]. *In vitro* experiments have confirmed that anti-LAG3 antibodies can downregulate Tregs, increase their cytotoxic activity against CD8^+^ T cells, reduce IFN-γ secretion, and modulate the immune tolerance of AML cells. Research has demonstrated that TIM3+ Treg cells exhibit elevated expression of inhibitory molecules, including LAG-3, PD-1, and CTLA-4, resulting in increased inhibitory function via releasing more IL-10, granzymes and perforin [52]. Dama et al. found that the high frequency of TIM-3+Tregs and Gal9+CD34-leukemic cells in the BM can promote T-cell exhaustion and induce immune escape in AML [53]. Therefore, targeting the Gal9/Tim-3 axis may improve AML patient prognosis. Additionally, research has indicated that functional single nucleotide polymorphisms (SNPs) of TIM-3 are connected to the risk prediction of AML [54]. The above studies revealed that high expression of coinhibitory molecules on Tregs can cause the dysfunction in APCs and effector T cells, promote their own differentiation and expansion, and are crucial for promoting immune escape and drug resistance in tumor cells (Figure 2). In summary, monitoring these immune-related biomarkers can help identify the immunosuppressive state in AML patients, predict treatment response, and provide guidance for personalized immunotherapy regimens.

Recently, the application of immune checkpoint inhibitors (ICIs) that target Tregs has provided new therapeutic directions and hope for overcoming resistance in AML. Widely studied and considered potential therapeutic targets include PD-1, TIM-3, LAG-3, and TIGIT. The clinical efficacy of ICIs is determined by factors, including the targeted pathway, disease stage, and combination with conventional therapies. Clinical studies have shown that when nivolumab or pembrolizumab is combined with azacitidine, the overall response rates (ORRs) are 33% and 55%, respectively, among individuals with relapsed/refractory (R/R) AML. Especially, patients who had not previously received hypomethylating agents (HMAs) had even higher ORRs [55–57], which may be related to the ability of HMA to increase the levels of PD-1/PD-L1 [58]. The combination of pembrolizumab with high-dose cytarabine or decitabine demonstrated efficacy, which was consistent with previous studies [59]. Other studies have shown that nonresponders to the combination of nivolumab and azacitidine primarily exhibit increased CTLA-4 expression on T lymphocytes [55]. The combination of ipilimumab with azacitidine and nivolumab for treating R/R AML demonstrated superior efficacy compared with the combination of nivolumab and azacitidine alone, along with an improvement in overall survival [60]. Research suggests that dual immunotherapy holds promising potential for clinical application. Additionally, research indicates that the application of ICIs targeting PD-1 (nivolumab) or CTLA-4 (ipilimumab) before stem cell transplantation (SCT) can enhance the progression-free survival (PFS) of AML/MDS patients post-transplant [61]. Clinical studies on the utilization of PD-L1 inhibitors for treating R/R AML have shown suboptimal therapeutic efficacy. For example, the combination of avelumab with decitabine has not yielded promising results [62]. The combination of atezolizumab with azacitidine has also shown limited therapeutic efficacy for treating R/R AML [63]. An earlier study showed that an anti-CTLA-4 mAb (MDX-010) induced the expansion of Teffs by enhancing the activity of DCs [64]. Research have demonstrated that the anti-TIGIT mAb TGTB227 is capable of suppressing the immunosuppressive function of Tregs by reducing the expression of Foxp3 in Tregs. This mechanism may be associated with the CD25/IL-2 signaling pathway [65]. Furthermore, an elevation in the frequency of TIGIT+ CD8^+^ T cells is associated with R/R AML and post-HSCT relapse. This effect is mediated primarily through the dysfunction of CD8^+^ T cells and diminished cytokine production. Moreover, knockdown of TIGIT can reverse the immunosuppressive effects induced by its high expression [66]. Tiragolumab, a novel anti-TIGIT monoclonal antibody, has demonstrated promising efficacy in studies on solid tumors when combined with the immune checkpoint inhibitor atezolizumab to overcome immunosuppression and restore immune responses [67]. Research on its use in malignant hematological tumors is limited. However, clinical studies on TIGIT inhibitors for R/R AML are relatively rare, although TIGIT inhibitors represent potential therapeutic targets for overcoming AML resistance. Sabatolimab (MBG453), a monoclonal antibody targeting TIM-3, has demonstrated the ability to enhance immune responses against leukemia cells *in vitro* [68]. We hypothesize that inhibiting the immune checkpoint TIM-3 may help alleviate the immunosuppressive effects of Treg cells and restore the antitumor immune activity of Teffs, making it a promising therapeutic approach for R/R AML. Currently, a study is underway to investigate whether sabatolimab, either as a monotherapy or combined with azacitidine, can amplify the graft-versus-leukemia (GvL) response in patients who achieve complete remission and are MRD positive following allogeneic stem cell transplantation (allo-SCT) [NCT04623216]. Relatlimab (BMS-986016), a human IgG4 anti-LAG-3 mAb, is a LAG-3-targeting drug that can enhance antitumor immune responses [69]. The combination of relatlimab with azacitidine in immune therapy for AML is currently under investigation [NCT04913922]. While LAG-3 inhibitors appear to be effective immunotherapeutic targets for treating R/R AML, further research is needed to increase their clinical applicability.

Tregs also express inducible T-cell costimulatory molecules (ICOSs) on their surface. Its binding to inducible T-cell costimulatory ligand (ICOSL) on AML cells can maintain overexpression of Foxp3 and CD25 and promote the transformation and proliferation of Tregs [70] (Figure 2). Moreover, the upregulation of IL-10 secretion by CD4^+^CD25+ICOS+ Tregs indicated that the expansion of Tregs in AML could be achieved through the ICOS/ICOSL pathway, which enhances the body’s immunosuppressive ability and induces leukemia cells to immune escape and drug resistance (Figure 2). Experiments have demonstrated that ICOS+ Tregs exhibit an increased suppressive effect on CD4^+^CD25^−^ effector T cells. Treatment of AML mice with anti-ICOSL mAb can decrease the number of Tregs, thereby slowing the progression of AML [70]. Thus, the strategy targeting the ICOS/ICOSL signaling pathways is expected to become new targets in drug-resistant AML. Elevated ICOS expression is linked to reduced OS rates in AML patients, and the coexpression of ICOS with PD-1 in non-M3 patients predicts even lower OS rates. Additionally, ICOS/PD-1 has emerged as an independent predictor of poor outcomes in AML [71]. Studies by Wan et al. have demonstrated that the proportion of Tregs in the BM of AML patients is greater than that observed in normal individuals, mainly because regulatory B cells (Bregs) induce CD4^+^CD25-T cells to transform into Tregs, and the chemokine receptor CXCR4 highly expressed on Tregs facilitates the strong migration and aggregation to the BM [72] (Figure 2). In addition, other chemokines, CCR1 and CCR5, expressed by Tregs can also cause Tregs to accumulate at the tumor site to mediate immunosuppression, promote immune evasion by cancer cells, and promote drug resistance recurrence. Animal experiments have confirmed that blocking the CXCL12-CXCR4 and CCL3-CCR1/CCR5 axes can inhibit the recruitment of Tregs in the bone marrow microenvironment and delay the progression of leukemia [73] (Figure 2), therefore, targeting the CXCL12-CXCR4 and CCL3-CCR1/CCR5 signaling pathways may become targets of leukemia immunotherapy. Moreover, AML cells can also effectively recruit Tregs by expressing CCL2, which binds to CCR2 receptors on Tregs [74]. These interactions help create an immunosuppressive microenvironment that enhances the survival and drug resistance of AML cells. Studies have shown that ICOSL, which is overexpressed on AML cells, interacts with ICOS+ Tregs, which enhances their ability to secrete IL-10, potentially inducing AML cell proliferation by triggering the Akt, Erk1/2, p38, and STAT3 signaling cascades. Additionally, it directly promotes the expansion of Tregs [70]. The upregulation of hypoxia-inducible factor (HIF) expression triggered by hypoxia in the AML microenvironment is associated with resistance to doxorubicin, possibly because HIF-1α enhances the expression of the YAP gene in AML cells, which stabilizes the binding of HIF-1α to its target genes [75]. Regulating the glycolytic pathway is associated with promoting Treg proliferation [76], further assisting in the evasion of immune surveillance by AML cells. Early studies have shown that leukemia-derived microvesicles (MVs) in AML patients suppress NKs cytotoxicity through TGF-β1, and this inhibition is mediated via the SMAD signaling pathway [27]. AML cell-derived extracellular vesicles (EVs) inhibit the activity of CD4^+^ T cells by carrying immune suppressive factors, such as PD-L1, TGF-β1 or miRNA, thereby promoting immune evasion in leukemia [77]. Research reported that leukemia cell-derived exosomes can stimulate bone marrow stromal cells (BMSCs) to secrete IL-8. IL-8 not only enhances the drug resistance of AML cells (e.g., etoposide) and promotes the survival of leukemia cells [78], but also interacts with the CXCR1 and CXCR2 on Tregs, facilitating the migration of Tregs to the tumor site and thereby suppressing effector T cells in the immune microenvironment. Hong et al.'s research indicated that EVs isolated from patients with relapsed/refractory AML can inhibit the antileukemic cytotoxicity of NK-92 cells [79]. These findings suggest that leukemia cells can induce the accumulation of Tregs at the tumor site and inhibit the antitumor immune response by changing the tumor immune microenvironment, thus affecting the outcome of chemotherapy and leading to drug resistance.

Over the past few years, substantial advancements have been achieved in AML immunotherapy, with the identification of multiple critical immune targets on AML cells that have been utilized for drug development. Among these, CD33 is a widely studied target, and its antibody‒drug conjugate, gemtuzumab ozogamicin, was approved in 2017 for use in treating newly diagnosed and relapsed or refractory CD33-positive AML patients [80, 81]. CD123, a marker of leukemia stem cells, has been targeted by drugs such as flotetuzumab (a CD123/CD3 bispecific antibody) and IMGN632 (an antibody‒drug conjugate), both of which have demonstrated therapeutic potential in R/R AML patients [82, 83]. The SIRPα-αCD123 fusion antibody targets both CD123 and CD47, with a specific focus on AML leukemia stem cells (LSCs). This dual-targeting approach significantly enhances immune clearance while reducing off-target toxicity, offering a novel strategy for achieving long-term remission and improved survival in AML patients [84]. These studies not only provide new strategies for the treatment of AML but also offer hope for improving patient survival rates and quality of life in the future.

## Other immune cells and drug resistance in AML

### Dendritic cells (DCs)

DCs originate from hematopoietic stem cells (HSCs) in the BM and are the most powerful APCs in the body. DCs are mainly categorized into conventional DCs (cDCs) and plasmacytoid DCs (pDCs). Mature cDCs, characterized by high expression of MHC II, the costimulatory molecules CD80/86 and CD40, and intercellular adhesion molecule 1 (ICAM-1), effectively present antigens, stimulate T cells, trigger adaptive immune responses, and fight tumors. pDCs also have the ability to process and present antigens, but their main function is to generate substantial amounts of type I interferon (IFN-I) and other proinflammatory factors, contribute to the antiviral innate immune response, and participate in the initiation and progression of tumors. Research have suggested that the expansion of pDCs is closely associated with the progression of AML [85]. The drug Tagraxofusp, which targets the pDC surface marker CD123, has shown significant efficacy in clearing pDCs [86]. Research by Wenbin et al. reported that the frequent occurrence of RUNX1 mutations is crucial for the differentiation and expansion of pDCs [86]. Therefore, CD123-targeted immunotherapy may represent a potential therapeutic approach for RUNX1-mutated AML. Importantly, Zhu et al. reported that patients with pDC infiltration in AML-M4/M5 patients had lower chemotherapy sensitivity and longer durations of CR and OS than those without pDC infiltration did, indicating that pDCs can be used for AML-M4/M5 risk stratification and to guide treatment [87]. Ocadlikova et al. reported that doxorubicin and cytarabine can induce the upregulation of CD39 or CD73 on DCs. The large amounts of ATP released by AML cells during exhaustion can be broken down by CD39 and CD73 into adenosine, which stabilizes the immune activity of Tregs, promoting the formation of an immunosuppressive microenvironment and inducing resistance [88]. ATP can bind to the P2X7 receptor on DCs to induce the upregulation of IDO1, depleting tryptophan and promoting the production of kynurenine, which inhibits T-cell proliferation and function [88–91]. Furthermore, Tregs release IL-10 and TGF-β, driving DCs and macrophages to adopt an immune-tolerant or protumor phenotype, further dampening the immune response [92, 93]. Other research has revealed that differentiation and proliferation of DCs rely on the FLT3/FLT3L signaling pathway, with FLT3 mutations being the most common in AML [94], and approximately 25% of patients harbor FLT3 mutations, which is linked to an unfavorable prognosis [95]. In recent years, with the development of tumor immunotherapy, DCs presenting tumor-specific antigens have been developed as vaccines, through which DCs present tumor antigens to T cells to induce adaptive immune responses to fight tumors and prevent drug resistance recurrence. However, the effectiveness of DC vaccination in inducing the anti-tumor response of immune system is relatively limited [96], and combined treatment with chemotherapy and radiotherapy may enhance the anti-tumor effect [97, 98]. Pepeldjiyska et al. reported that the increase in the frequency of leukemia-derived DCs (DCleu) derived from bone marrow primitive leukemia cells in AML patients can downregulate Tregs and activate specific T cells and NKs, promoting anti-tumor immune response [99], thus, the heightened frequency of induced DCleu helps to increase the body’s antitumor immunity and reverse tumor resistance.

### Natural killer cells (NKs)

NKs are lymphoid innate immune cells and are the key effector cells in immunotherapy [100], which are related to the occurrence, progression and recurrence of AML [101]. The killer cell immunoglobulin-like receptor (KIR) expressed on the surface of NKs can be divided into two types, inhibitory and activating [102], which can specifically recognize and bind target cell surface molecules and play important antitumor roles [103]. NKs express different immunophenotypes at the immature, mature, and hypermature stages and can migrate, release cytokines, and destroy target cells [104]. A study reported that the ratio of NK cells in the BM of newly diagnosed AML patients may forecast patient outcomes. In comparison to healthy individuals, the proportion of NKs in the BM of R/R patients is the lowest [105], and their function is impaired, suggesting that a reduced NK cell ratio correlates with a worse prognosis in AML patients [106, 107]. NK-based immunotherapy can significantly improve the outcomes of patients with advanced or high-risk AML [108]. Studies have shown that the overexpression of heme oxygenase 1 (HO1) in AML patients induces NK dysfunction [109], thus, targeting HO1 to restore NK cell function may be a promising anti-AML immunotherapy strategy. Dai et al. reported that the myeloid cell leukemia-1 (MCL1) is negatively correlated with that of NKs and that the combined action of MCL1 inhibitors and NKs can significantly exhaust primary AML cells and cell lines. Interestingly, the proportion of NKs in the BM can affect the effect of MCL1 inhibitors [105], indicating that the proportion and abnormal function of NKs play important roles in the drug resistance of AML leukemia cells. Chajuwan et al. observed that elevated TIM-3 expression in NKs correlated with CR status after induction therapy, indicating that TIM-3 in NKs may be a prognostic marker for AML [110]. Bou-Tayeh et al. reported that NKs from mice with leukemia express IL-15/mTOR signaling, and this pathway can induce NK metabolism and functional failure in leukemic mice [111]. Compared with that in healthy controls, AML patients showed reduced levels of the NK-activating receptors NKG2D, NKp46, and NKp30 in their peripheral blood, while there was an upregulation in the expression of inhibitory receptors such as TIM-3, ILT-4, ILT-5, and PD-1. At the same time, the expression of Siglec-7 in NK cells was notably reduced in AML patients [101], suggesting that Siglec7 is an indicator of NK cell function and could potentially be targeted to improve NK cell activity, thereby boosting the antitumor immune response. Disruption of the NKG2D/NKG2DL pathway, which is crucial for NK cell-mediated tumor cell killing, can lead to immune escape in AML, resulting in drug resistance [112]. Furthermore, NKs can release cytokines like IFN-γ and perforin/granzyme, and express TNF-related apoptosis-inducing ligand (TRAIL) and Fas Ligand (FasL) to activate apoptotic pathways in tumor cells [113]. The loss of these functions may represent a key mechanism through which tumors escape killing mediated by NK cells. The immunosuppressive tumor microenvironment created by TAMs, MDSCs, and Tregs is a major obstacle to NK cell-mediated antitumor activity. They drive NKs exhaustion and facilitate tumor immune escape by depleting activation factors like IL-2, releasing immunosuppressive molecules such as TGF-β and IL-10, and activating inhibitory receptors on NK cells, including PD-1 and TIGIT. Studies have shown that NK cells recognize ligands like PDL1, Gal-9, and CD112/CD155 on AML cells through their surface receptors PD-1, TIM3, and TIGIT, triggering inhibitory signaling pathways. This activation affects NK cell function via the PI3K, ERK, and PKCθ pathways, thereby facilitating tumor immune evasion [114, 115]. Moreover, the high expression of CD200 on leukemia cells, through binding to CD200R, inhibits the cytotoxicity of NK cells, inducing immune evasion of leukemia cells, which is associated with the recurrence and progression of AML [116, 117]. NKs exhibit strong anti-tumor activity. Restoring the antitumor properties of NK cells may represent a promising approach for treating relapse. Notably, AML cells can evade the immune recognition of NKs via gene mutation, fusion and epigenetic modification, though the exact mechanism remains uncertain [118]. Research has shown that the hypomethylation agent (HMA) decitabine can upregulate the level of ICAM-1(CD54) and CD48 on AML cells, thereby activating NKs to kill leukemia cells while reversing immune evasion by leukemia cells [118, 119]. This suggests that the combination of HMA and NK cells infusion may serve as a promising new approach for AML treatment.

The immune exhaustion of effector immune cells poses a critical challenge in cancer treatment. Current strategies to reverse T cells and NKs exhaustion primarily include ICIs, activation receptor enhancement, and the application of genetically engineered CAR-T/CAR-NK cells. ICIs target molecules such as PD-1, CTLA-4, TIM-3, TIGIT, and LAG-3, blocking the interaction of checkpoint molecules to relieve the inhibition of effector immune cells, enhance their activity, reverse immune exhaustion, and overcome immune resistance. IL-2 and IL-15 are critical cytokines that promote the growth and activation of T cells and NK cells [120, 121]. Local or systemic administration of recombinant IL-2 or IL-15 can enhance the proliferation and function of immune cells, thereby reversing immune exhaustion. Additionally, a study on CD33-targeted CAR-T-cell therapy for AML demonstrated a transient reduction in CD33^+^ leukemic blasts (lasting only 7 days), accompanied by adverse effects such as leukopenia [122]. CD123-targeted CAR-T-cell therapy can achieve CR in AML patients, but it is linked to notable adverse effects [123, 124]. NKG2D is a type II transmembrane receptor, and its signaling induces cytolytic effector functions [125]. In a study involving 22 AML patients, repeated infusions of NKG2D CAR-T cells resulted in a 4.5% probability of achieving a morphologic leukemia-free state (MLFS) [126]. CAR-T-cell therapy for AML remains under study but has demonstrated significant potential. Owing to the potent antitumor properties of NK cells, CAR-NK cells can also specifically target tumor cells [127], and CAR-NK cells offer several advantages, including immediate availability, inducible proliferation, and a longer lifespan [128]. However, when donor-derived NKs are used, GVHD may occur [129].

### Cytotoxic T lymphocytes (CTLs)

CD8^+^ T cells are activated, proliferate, and differentiate into CTLs in peripheral immune organs and can accumulate at tumor sites under the action of chemokines. The surface of CD8^+^ T cells highly expresses lymphocyte function-associated antigen-1 (LFA-1) and CD2, which can interact with the expression of ICAM-1. LFA-3 binds to target cells and kills target cells efficiently. Early research has demonstrated that the accumulation of Tregs in the AML progression model can suppress the expansion of CTLs [130], potentially through the secretion of the inhibitory cytokines IL-10 and TNF-β. Leukemic progenitor cells and leukemic stem cells (LPCs/LSCs) are currently believed to be responsible for disease relapse after intensive therapy, and targeting LPCs/LSCs through specific CTLs may be an option to prevent AML relapse [131]. The application of anti-PD-1 antibody (nivolumab) in AML significantly increases T-cell-directed immune response targeting leukemia-associated antigen (LAA), particularly in the context of targeting LPCs [131], thus, the nivolumab could a candidate immunotherapy for those who are resistant. However, Rakova reported that ICIs targeting PD-1 and CTLA4 exhibit limited clinical effectiveness in AML [132]. In AML with TP53 gene mutations, TIM-3 expression is significantly increased, and CTLs exhibit characteristics of exhaustion/dysfunction, indicating that the antitumor immune response of TP53-mutated AML is insufficient, which presents a new strategy for overcoming drug resistance in AML [133]. Studies have suggested that IFN-I can promote the recruitment of tumor-specific CTLs; therefore, stimulator of interferon genes (STING) agonists have a killing effect on AML leukemia cells [134]. In conclusion, CTLs specifically kill target cells, and immunotherapy to restore and enhance the cytotoxic function of CTLs is an effective treatment to combat tumor drug resistance.

A clinical study demonstrated progress in treating relapsed AML patients with tumor-associated antigen-specific cytotoxic T lymphocytes (TAA-CTLs), showing that some patients achieved MRD negativity with a reduced incidence of relapse [135], but the study involved a limited sample size, leading to lack statistical significance and reliability. However, some studies have revealed that immunotherapeutic approaches with TAA-CTLs are less reliable at eradicating the disease, potentially because of the immunosuppressive tumor microenvironment [136]. Studies indicate that LAA-CTLs (such as CG1-CTLs or PR1-CTLs) combined with pembrolizumab are more effective in eliminating AML cells than LAA-CTLs alone without increasing toxic side effects or the risk of GVHD [136, 137]. Chapuis et al. used adoptive transfer of WT1-specific allogeneic TCR-T cells in a clinical trial to treat high-risk AML patients, achieving good therapeutic outcomes and helping to prevent disease relapse (NCT01640301) [138]. In addition, an additional clinical study of autologous WT1-specific TCR-T cells aimed to evaluate their therapeutic effects on high-risk bone marrow malignancies, which showed significant efficacy and excellent safety (NCT02550535) [139].

### T helper 17 (Th17) cells

**FIGURE 3:**
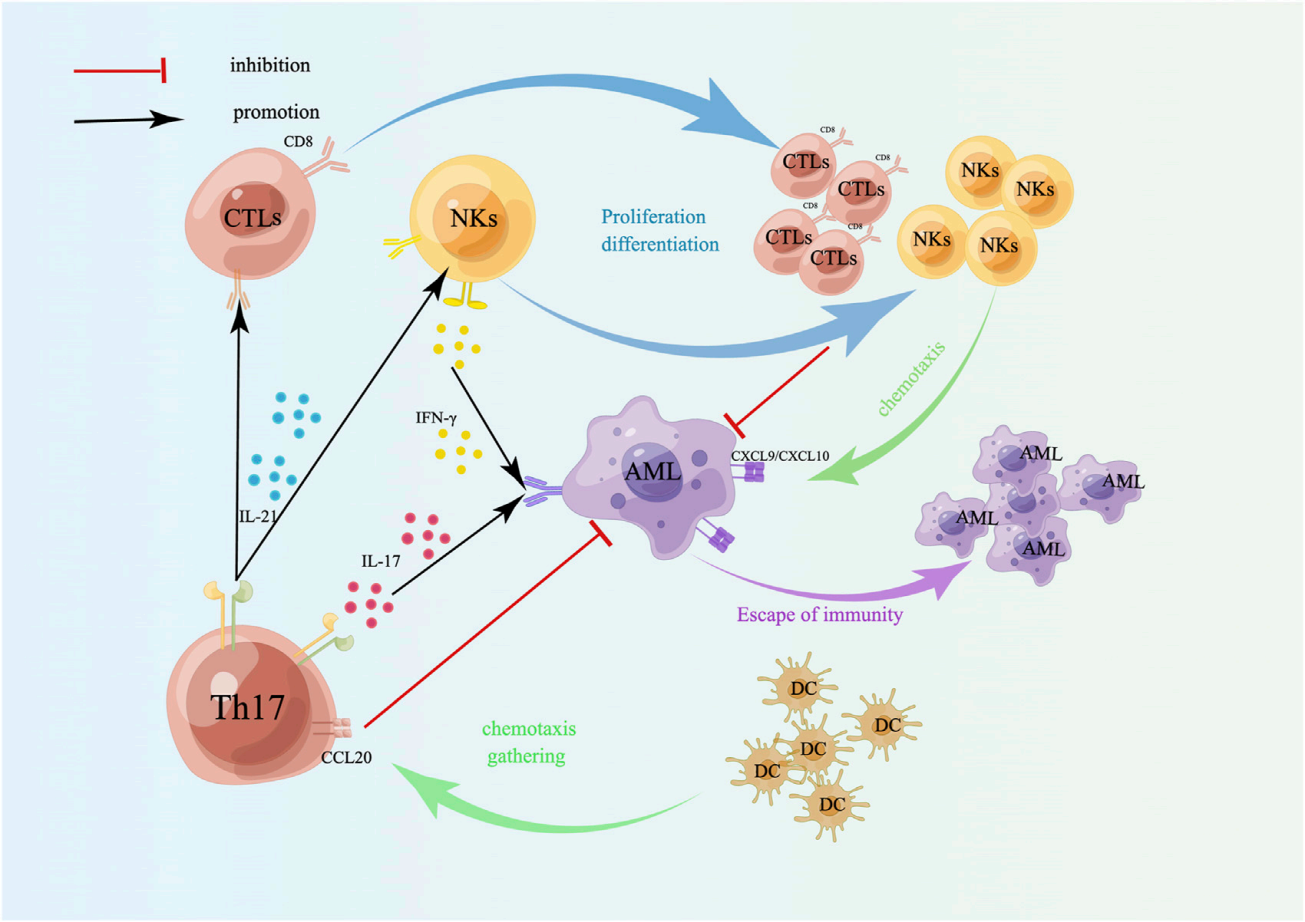
Targeting effect of Th17 cells on AML cells. Th17 cells induce the differentiation and proliferation of CTLs and NKs by secreting IL-21; IL-17 secreted by Th17 cells and IFN-γ secreted by NKs act on AML cells together, which can induce tumor cells to secrete CXCL9/CXCL10, accumulate NKs and CTLs, and kill tumor cells. Th17 cells also recruit DCs by releasing CCL20 to enhance the immune response at the tumor site. A schematic diagram is created by Figdraw (www.figdraw.com).

**FIGURE 4:**
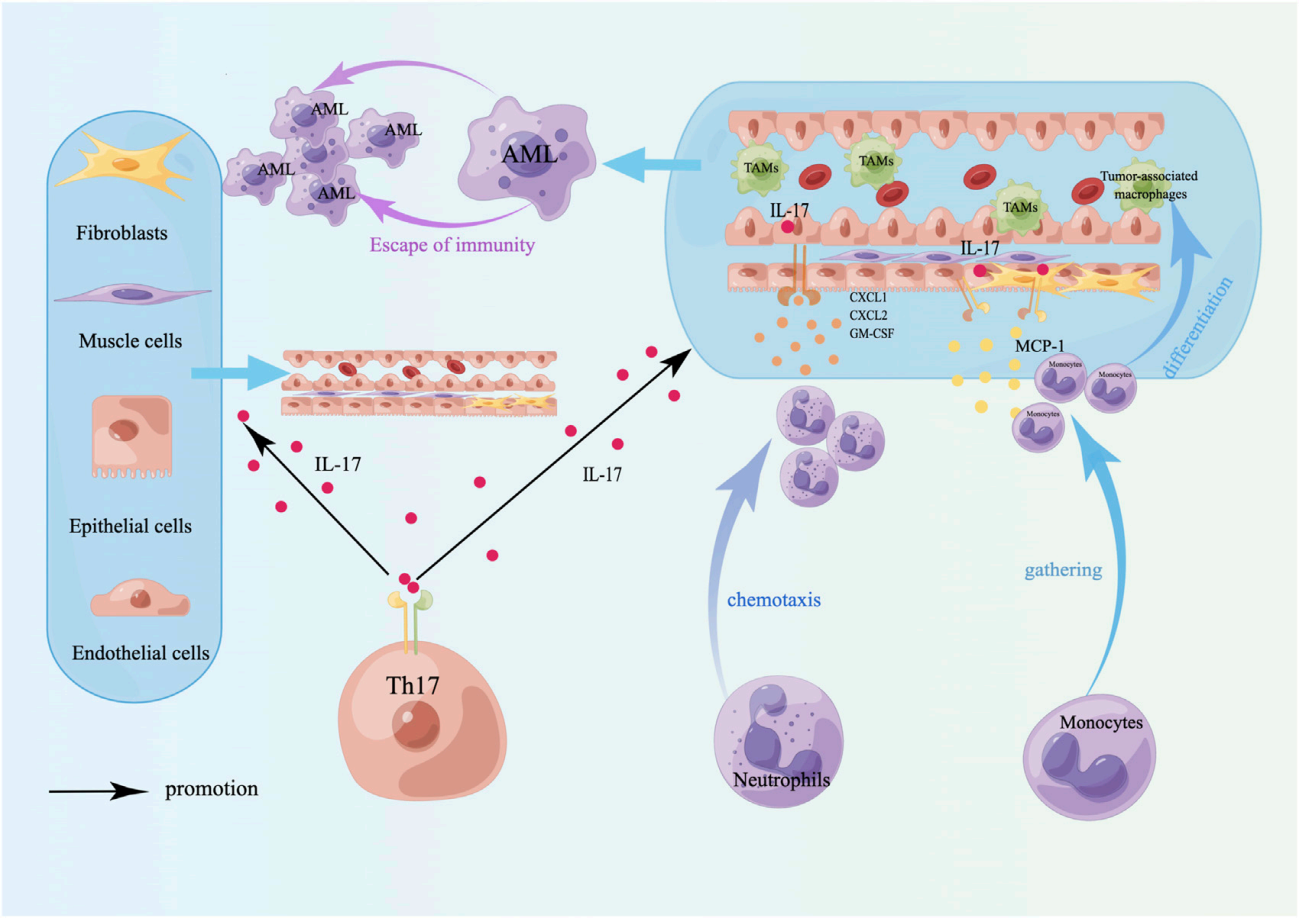
Tumor-promoting effects of Th17 cells. The tumor-promoting effect of Th17 cells is mainly to induce angiogenesis and TAM differentiation. TAMs lose their antitumor properties, have immunosuppressive effects, induce immune escape of tumor cells, and are linked to a poor prognosis in AML. A schematic diagram is created by Figdraw (www.figdraw.com).

The primary function of Th17 cells is to induce neutrophils to phagocytose and kill pathogens, primarily by secreting IL-17, IL-21, and IL-22 to exert their immune effects. Among these cytokines, IL-21 can amplify Th17 function through autocrine signaling, stimulate CD8^+^ T-cell and NK proliferation and differentiation, and have antitumor immune effects (Figure 3). IL-17 has both tumor-promoting and antitumor effects. The tumor-promoting properties of AML are mediated mainly by angiogenesis, which stimulates endothelial cells to release chemokines (CXCL1, CXCL2) and growth factors (GM-CSF) to recruit neutrophils, and induces epithelial cells and fibroblasts to release monocyte chemoattractant protein 1 (MCP-1) to recruit monocytes to tumor sites to differentiate into TAMs [140], whereas TAMs lose their antitumor immune properties and are linked to unfavorable outcomes [141] (Figure 4). The antitumor effect of IL-17 is synergistic with that of IFN-γ, which stimulates tumor cells to release the chemokines CXCL9 and CXCL10 to recruit NKs and CTLs into tumor sites [140, 142] (Figure 3). In addition, Th17 cells can also recruit DCs by releasing CCL20, which may enhance the immune response at the tumor site [140] (Figure 3). Studies have confirmed that the proportion of Th17 cells and the concentration of IL-17 in the bone marrow of patients with newly diagnosed and relapsed AML are significantly increased and that there is no notable distinction between these patients and healthy controls in the CR and disease-free survival (DFS) stages [143]. Ren et al. reported that the overexpression of beta-1,4-galactosyltransferase 1 (B4GALT1) in AML may be linked to poor patient outcomes, and the proportion of Th17 cells shows a positive correlation with B4GALT1 levels [144]. In newly diagnosed AML patients, peripheral blood CD4^+^ T cells (mainly Th17 cells) secrete abundant TNF-α, which binds to TNFR2 on the surface of Tregs, inducing Treg expansion and enhancing their function [145]. An *in vitro* study confirmed that IL-1β, IL-6 and IL-23 can promote naive CD4^+^ T cells to differentiate into Th17 cells. High levels of Th17 cells can promote the proliferation and poor prognosis of AML patients through IL-17-mediated activation of the PI3K/AKT and JAK/STAT3 pathways. In addition, high levels of Th17 cells can inhibit Th1 cell and IFN-γ production through the secretion of IL-17 and IL-22 [146]. Another clinical study indicated that the cytokines IL-23 and IL-17 secreted by Th17 cells are also positively correlated with poor clinical outcomes in AML [147].

This novel epigenetic therapy shows safety and efficacy in managing early relapse in non-APL AML patients following transplantation, with Th1/Th17 ratio modulation offering both immunological benefits and potential as a biomarker for relapse monitoring [148]. The traditional stimulator of interferon genes pathway can effectively improve antitumor immunity. However, owing to its easy degradation and membrane transport difficulties, its antitumor effect is blocked. Emerging bioinspired nanomedicines can enhance the STING pathway to target AML cells systemically, and the mechanism may be related to the increased proportion of Th1/Th17 cells, the concentration of IFN-I and proinflammatory cytokines, and the decreased proportion of Th2 cells [149]. Elevated expression of the immunoregulatory transcription factor ZEB1 is related to poor overall survival, and the underlying mechanism may involve the promotion of Th17 cell development, increasing the secretion of IL-17 and TGF-β, and the expression of suppressor of cytokine signaling 2 (SOCS2) [150]. Therefore, ZEB1 may be a promising therapeutic target for AML.

### Myeloid-derived suppressor cells (MDSCs)

MDSCs are composed mainly of granulocyte myeloid-derived suppressor cells (G-MDSCs, CD66b+CD15+HLA-DR- cells), monocyte MDSCs (M-MDSCs, CD14^+^CD11b+CD33+HLA-DR-/lo cells) and immature myeloid cells (IMCs, CD11b+CD33^+^CD14-HLA-DR-CD34^+^ cells) [151]. An increase in the number of MDSCs in myeloid malignancies results in a significant immunosuppressive effect, which may induce immune escape of tumor cells and promote tumor development [152]. MDSCs can inhibit Teffs by releasing arginase 1 (Arg1), nitric oxide synthase 2 (NOS2), reactive oxygen species (ROS), cyclooxygenase 2 (COX2), TGF-β, etc. [153–155], thereby promoting the progression of various cancers. In addition, MDSCs can indirectly upregulate Tregs [156]. In AML, MDSCs can suppress potent antitumor immune responses [157] and can suppress the function of CD8^+^ T cells via high expression of Arg1 and indoleamine-2,3-dioxygenase 1 (IDO) [158]. Among them, IDO1 can decompose tryptophan, and a decrease in tryptophan and the accumulation of its metabolites can suppress the proliferation of antigen-specific T cells and trigger their apoptosis [159]. Arginase II induces T-cell apoptosis and autophagy by depriving T cells of the ability to metabolize the essential amino acid arginine [160], thereby reducing the immune effect of T cells on cancer cells. MDSCs and Tregs are complex, as they can enhance their interactions through soluble mediators (such as IL-10 and TGF-β), metabolic cooperation (such as Arg-1, iNOS, and CD73), and intercellular communication (such as PD-L1/PD-1, CD80/CTLA-4, and CD40/CD40L), establishing a complex immunosuppressive feedback mechanism [161]. Ren et al. reported that high levels of M-MDSCs, which possess potent immunosuppressive capabilities, can predict poor prognosis in AML patients [162]. In addition, Tohumeken et al. reported that AML-derived EVs can induce conventional monocytes to differentiate into MDSCs, obtain a CD14+HLADRlow phenotype, and upregulate IDO to inhibit effector T-cell immunity. The Akt/mTOR pathway plays a critical factor in the phenotype and functional transformation of monocytes induced by AML-released EVs [163], and MDSCs also significantly inhibit the proliferation of NKs [164], indicating that an increase in MDSCs facilitates tumor cell immune escape. Research by Bai et al. revealed that the mechanism of AraC-induced AML resistance may involve the amplification of TNF-α, which activates the IL-6/STAT3 and NF-KB pathways, enhancing the function and survival of MDSCs and thereby mediating the immune evasion by tumors and drug resistance [165], suggesting that chemotherapy combined with TNF-α-targeting therapy may be an effective strategy to inhibit MDSC-induced immune escape. Additionally, Pyzer et al. reported that AML induces the release of the c-Myc protein through MUC1-C signaling, which inhibits the expression of miR34a, driving the proliferation of MDSCs, the levels of PD-L1, and immunosuppressive functions [166]. These findings highlight the potential of targeting the MUC1-C/c-Myc pathway as an approach for AML immunotherapy. Au et al. reported that the accumulation of immunosuppressive cells, including Tregs, MDSCs (mainly G-MDSCs) and TAMs, in the BM of AML patients is connected to CD34^+^ AML progenitor cells [32], which promotes the immune evasion of AML blasts. Research has demonstrated that MDSC-like progenitor cells can induce Tregs expansion and cause CTLs dysfunction and exhaustion [158, 167], further reinforcing the tumor-suppressive microenvironment, which is closely associated with relapse following allo-HSCT in AML [168]. VISTA inhibits the activity of CD8^+^ T cells via high expression on MDSCs from AML patients, and its synergistic effect with PD-1 suggests that combined inhibition of VISTA and PD-1 pathways may be a new strategy to enhance AML immunotherapy [169]. *In vitro* studies have shown that an IDO1 inhibitor (INCB024360) can induce the proliferation of CTLs, reduce Tregs, and decrease the immunosuppressive activity of MDSCs [170]. However, a phase II trial in MDS patients revealed that INCB024360 had no significant therapeutic effect [171]. Currently, research on INCB024360 (epacadostat) in AML is relatively limited. Research conducted by Masahiro et al. showed that DC vaccines loaded with Wilms’ tumor 1 (WT1) enhance immune surveillance by reducing the number of MDSCs and downregulating their immunosuppressive functions, particularly the arginase 1 and IDO pathways, offering new hope for immunotherapy in AML [172]. Miner et al.'s study revealed that myeloid leukemia cells (including AML and MDS cells) can inhibit T-cell function through the STAT3 and arginase pathways [173]. These findings suggest that therapeutic strategies targeting STAT3 and arginase inhibitors may increase the effectiveness of leukemia immunotherapy and improve immune evasion mechanisms.

## Summary

We conducted a detailed exploration of the immune mechanisms responsible for resistance to treatment in AML, with an emphasis on immune cells in the bone marrow microenvironment. Research indicates that Tregs contribute significantly to immune evasion and chemoresistance by releasing inhibitory cytokines and expressing immune checkpoint molecules, with elevated Treg levels being associated with poor prognosis. AML cells further promote tumor survival and evade immune surveillance by releasing immunosuppressive molecules and altering the bone marrow microenvironment. DCs, NKs, and CTLs also contribute significantly to immune regulation in AML, with impaired DC function, suppressed NK activity, and reduced CTL antitumor capacity closely linked to drug resistance. Moreover, myeloid-derived suppressor cells exacerbate immune suppression by secreting metabolic inhibitory molecules and increasing Treg activity. We also summarize recent advances in immunotherapy, including ICIs, CAR-T and CAR-NK cell therapies, and treatments targeting AML-specific antigens, all of which have potential in enhancing immune function and overcoming drug resistance. We emphasize the need for future research to optimize immunotherapy protocols, integrate chemotherapy and other treatments, improve patient outcomes and increase survival rates.

## References

[1] Niu J Peng D Liu L. Drug resistance mechanisms of acute myeloid leukemia stem cells. Front Oncol (2022) 12:896426. 10.3389/fonc.2022.896426 35865470 PMC9294245

[2] Shapoorian H Zalpoor H Ganjalikhani-Hakemi M. The correlation between Flt3-ITD mutation in dendritic cells with TIM-3 expression in acute myeloid leukemia. Blood Science Baltimore, Md (2021) 3(4):132–5. 10.1097/bs9.0000000000000092 35402842 PMC8975045

[3] Conneely SE Stevens AM. Acute myeloid leukemia in children: emerging paradigms in genetics and new approaches to therapy. Curr Oncol Rep (2021) 23(2):16. 10.1007/s11912-020-01009-3 33439382 PMC7806552

[4] Buckley SA Kirtane K Walter RB Lee SJ Lyman GH. Patient-reported outcomes in acute myeloid leukemia: where are we now? Blood Rev (2018) 32(1):81–7. 10.1016/j.blre.2017.08.010 28888621

[5] Song Y Wang N Chen L Fang L. Tr1 cells as a key regulator for maintaining immune homeostasis in transplantation. Front Immunol (2021) 12:671579. 10.3389/fimmu.2021.671579 33981317 PMC8109434

[6] Carrier Y Yuan J Kuchroo VK Weiner HL. Th3 cells in peripheral tolerance. I. Induction of foxp3-positive regulatory T cells by Th3 cells derived from TGF-β T cell-transgenic mice. The J Immunol (2007) 178(1):179–85. 10.4049/jimmunol.178.1.179 17182553

[7] Davidson TS DiPaolo RJ Andersson J Shevach EM. Cutting edge: IL-2 is essential for TGF-β-mediated induction of Foxp3+ T regulatory cells. The J Immunol (2007) 178(7):4022–6. 10.4049/jimmunol.178.7.4022 17371955

[8] Wang W Hong T Wang X Wang R Du Y Gao Q Newly found peacekeeper: potential of CD8+ Tregs for graft-versus-host disease. Front Immunol (2021) 12:764786. 10.3389/fimmu.2021.764786 34899714 PMC8652293

[9] Ford MS Zhang ZX Chen W Zhang L. Double-negative T regulatory cells can develop outside the thymus and do not mature from CD8+ T cell precursors. The J Immunol (2006) 177(5):2803–9. 10.4049/jimmunol.177.5.2803 16920915

[10] Thomson CW Lee BP Zhang L. Double-negative regulatory T cells: non-conventional regulators. Immunologic Res (2006) 35(1-2):163–78. 10.1385/ir:35:1:163 17003518

[11] Su Y Huang X Wang S Min WP Yin Z Jevnikar AM Double negative Treg cells promote nonmyeloablative bone marrow chimerism by inducing T-cell clonal deletion and suppressing NK cell function. Eur J Immunol (2012) 42(5):1216–25. 10.1002/eji.201141808 22539294

[12] McIver Z Wlodarski M Powers J O’Keefe C Jin T Sobecks R Double negative T cells influence TCR VB variablity to induce allotolerance. Blood (2006) 108(11):759–9. 10.1182/blood.v108.11.759.759

[13] Ford McIntyre MS Gao JF Li X Naeini BM Zhang L. Consequences of double negative regulatory T cell and antigen presenting cell interaction on immune response suppression. Int immunopharmacology (2011) 11(5):597–603. 10.1016/j.intimp.2010.11.015 21109036

[14] Achita P Dervovic D Ly D Lee JB Haug T Joe B Infusion of *ex-vivo* expanded human TCR-αβ+ double-negative regulatory T cells delays onset of xenogeneic graft-versus-host disease. Clin Exp Immunol (2018) 193(3):386–99. 10.1111/cei.13145 30066399 PMC6150261

[15] Ono M. Control of regulatory T-cell differentiation and function by T-cell receptor signalling and Foxp3 transcription factor complexes. Immunology (2020) 160(1):24–37. 10.1111/imm.13178 32022254 PMC7160660

[16] Zhang Y Maksimovic J Naselli G Qian J Chopin M Blewitt ME Genome-wide DNA methylation analysis identifies hypomethylated genes regulated by FOXP3 in human regulatory T cells. Blood (2013) 122(16):2823–36. 10.1182/blood-2013-02-481788 23974203 PMC3798997

[17] Szczepanski MJ Szajnik M Czystowska M Mandapathil M Strauss L Welsh A Increased frequency and suppression by regulatory T cells in patients with acute myelogenous leukemia. Clin Cancer Res (2009) 15(10):3325–32. 10.1158/1078-0432.ccr-08-3010 19417016 PMC3700356

[18] Yang PX Wang P Fang L Wang Q. Expression of Tregs and IL-35 in peripheral blood of patients with newly diagnosed acute myeloid leukemia. Zhongguo shi yan xue ye xue za zhi (2022) 30(6):1688–92. 10.19746/j.cnki.issn.1009-2137.2022.06.010 36476890

[19] Ahmed HA Maklad AM Khaled SA Elyamany A. Interleukin-27 and interleukin-35 in *de novo* acute myeloid leukemia: expression and significance as biological markers. J Blood Med (2019) 10:341–9. 10.2147/jbm.s221301 31686937 PMC6783395

[20] Wang J Tao Q Wang H Wang Z Wu F Pan Y Elevated IL-35 in bone marrow of the patients with acute myeloid leukemia. Hum Immunol (2015) 76(9):681–6. 10.1016/j.humimm.2015.09.020 26431888

[21] Chen W Jin W Hardegen N Lei KJ Li L Marinos N Conversion of peripheral CD4+CD25− naive T cells to CD4+CD25+ regulatory T cells by TGF-β induction of transcription factor *Foxp3* . The J Exp Med (2003) 198(12):1875–86. 10.1084/jem.20030152 14676299 PMC2194145

[22] Tao Q Pan Y Wang Y Wang H Xiong S Li Q Regulatory T cells-derived IL-35 promotes the growth of adult acute myeloid leukemia blasts. Int J Cancer (2015) 137(10):2384–93. 10.1002/ijc.29563 25866142

[23] Shenghui Z Yixiang H Jianbo W Kang Y Laixi B Yan Z Elevated frequencies of CD4^+^CD25^+^CD127^lo^ regulatory T cells is associated to poor prognosis in patients with acute myeloid leukemia. Int J Cancer (2011) 129(6):1373–81. 10.1002/ijc.25791 21105040

[24] Tan J Chen S Xu L Lu S Zhang Y Chen J Increasing frequency of T cell immunosuppressive receptor expression in CD4+ and CD8+ T cells may related to T cell exhaustion and immunosuppression in patients with AML. Blood (2016) 128(22):5166–6. 10.1182/blood.v128.22.5166.5166

[25] Sanchez-Correa B Bergua JM Campos C Gayoso I Arcos MJ Bañas H Cytokine profiles in acute myeloid leukemia patients at diagnosis: survival is inversely correlated with IL-6 and directly correlated with IL-10 levels. Cytokine (2013) 61(3):885–91. 10.1016/j.cyto.2012.12.023 23357299

[26] Rashed R Shafik RE Shafik NF Shafik HE. Associations of interleukin-10 gene polymorphisms with acute myeloid leukemia in human (Egypt). J Cancer Res Ther (2018) 14(5):1083–6. 10.4103/0973-1482.187367 30197353

[27] Boyiadzis M Szczepanski M Whiteside T. Membrane associated TGF-β1 on leukemia blast-derived microvesicles in sera of acute myeloid leukemia patients suppresses NK cell function. Blood (2010) 116(21):502–2. 10.1182/blood.v116.21.502.502

[28] Zhang Q Huang T Li X Liu G Xian L Mao X Prognostic impact of enhanced CD96 expression on NK cells by TGF-β1 in AML. Int immunopharmacology (2024) 141:112958. 10.1016/j.intimp.2024.112958 39159564

[29] Reichelt P Bernhart S Wilke F Schwind S Cross M Platzbecker U MicroRNA expression patterns reveal a role of the TGF-β family signaling in AML chemo-resistance. Cancers (2023) 15(20):5086. 10.3390/cancers15205086 37894453 PMC10605523

[30] Wang D Sun Z Zhu X Zheng X Zhou Y Lu Y GARP-mediated active TGF-β1 induces bone marrow NK cell dysfunction in AML patients with early relapse post–allo-HSCT. Blood (2022) 140(26):2788–804. 10.1182/blood.2022015474 35981475 PMC10653097

[31] Han S Wang W Wang S Yang T Zhang G Wang D Tumor microenvironment remodeling and tumor therapy based on M2-like tumor associated macrophage-targeting nano-complexes. Theranostics (2021) 11(6):2892–916. 10.7150/thno.50928 33456579 PMC7806477

[32] Au Q Hanifi A Parnell E Kuo J Leones E Sahafi F Phenotypic characterization of the immune landscape in the bone marrow of patients with acute myeloid leukemia (AML) using MultiOmyxTM hyperplexed immunofluorescence assay. Blood (2019) 134(Suppl. 1):1455–5. 10.1182/blood-2019-122206

[33] Bachanova V Cooley S Defor TE Verneris MR Zhang B McKenna DH Clearance of acute myeloid leukemia by haploidentical natural killer cells is improved using IL-2 diphtheria toxin fusion protein. Blood (2014) 123(25):3855–63. 10.1182/blood-2013-10-532531 24719405 PMC4064329

[34] Cooley S Bachanova V Geller M Verneris M Zhang B McCullar V IL-2 stimulated treg inhibit *in vitro* expansion of haploidentical natural killer (NK) cells, which is partially overcome with an IL-2-diphtheria toxin fusion protein *in vivo* . Blood (2011) 118(21):3611–1. 10.1182/blood.v118.21.3611.3611

[35] Frimpong-Boateng K van Rooijen N Geiben-Lynn R. Regulatory T cells suppress natural killer cells during plasmid DNA vaccination in mice, blunting the CD8+ T cell immune response by the cytokine TGFβ. PloS one (2010) 5(8):e12281. 10.1371/journal.pone.0012281 20808850 PMC2924372

[36] Qureshi OS Zheng Y Nakamura K Attridge K Manzotti C Schmidt EM Trans-endocytosis of CD80 and CD86: a molecular basis for the cell-extrinsic function of CTLA-4. Science (2011) 332(6029):600–3. 10.1126/science.1202947 21474713 PMC3198051

[37] Radwan SM Elleboudy NS Nabih NA Kamal AM. The immune checkpoints Cytotoxic T lymphocyte antigen-4 and Lymphocyte activation gene-3 expression is up-regulated in acute myeloid leukemia. Hla (2020) 96(1):3–12. 10.1111/tan.13872 32189430

[38] El Dosoky W Aref S El Menshawy N Ramez A Abou Zaid T Aref M Prognostic effect of CTLA4/LAG3 expression by T-cells subsets on acute myeloid leukemia patients. Asian Pac J Cancer Prev (2024) 25(5):1777–85. 10.31557/apjcp.2024.25.5.1777 38809650 PMC11318815

[39] Ramzi M Iravani Saadi M Yaghobi R Arandi N. Dysregulated expression of CD28 and CTLA-4 molecules in patients with acute myeloid leukemia and possible association with development of graft versus host disease after hematopoietic stem cell transplantation. Int J Organ Transplant Med (2019) 10(2):84–90. 31285805 PMC6604755

[40] Davids MS Kim HT Bachireddy P Costello C Liguori R Savell A Ipilimumab for patients with relapse after allogeneic transplantation. New Engl J Med (2016) 375(2):143–53. 10.1056/nejmoa1601202 27410923 PMC5149459

[41] Dong Y Han Y Huang Y Jiang S Huang Z Chen R PD-L1 is expressed and promotes the expansion of regulatory T cells in acute myeloid leukemia. Front Immunol (2020) 11:1710. 10.3389/fimmu.2020.01710 32849603 PMC7412746

[42] Davis KL Agarwal AM Verma AR. Checkpoint inhibition in pediatric hematologic malignancies. Pediatr Hematol Oncol (2017) 34(6-7):379–94. 10.1080/08880018.2017.1383542 29190182

[43] Chen C Liang C Wang S Chio CL Zhang Y Zeng C Expression patterns of immune checkpoints in acute myeloid leukemia. J Hematol and Oncol (2020) 13(1):28. 10.1186/s13045-020-00853-x 32245463 PMC7118887

[44] Zhang Y Huang Y Hong Y Lin Z Zha J Zhu Y Lactate acid promotes PD-1+ Tregs accumulation in the bone marrow with high tumor burden of Acute myeloid leukemia. Int immunopharmacology (2024) 130:111765. 10.1016/j.intimp.2024.111765 38447414

[45] Joller N Lozano E Burkett PR Patel B Xiao S Zhu C Treg cells expressing the coinhibitory molecule TIGIT selectively inhibit proinflammatory Th1 and Th17 cell responses. Immunity (2014) 40(4):569–81. 10.1016/j.immuni.2014.02.012 24745333 PMC4070748

[46] Triebel F Jitsukawa S Baixeras E Roman-Roman S Genevee C Viegas-Pequignot E LAG-3, a novel lymphocyte activation gene closely related to CD4. The J Exp Med (1990) 171(5):1393–405. 10.1084/jem.171.5.1393 1692078 PMC2187904

[47] Anderson AC Joller N Kuchroo VK. Lag-3, tim-3, and TIGIT: Co-inhibitory receptors with specialized functions in immune regulation. Immunity (2016) 44(5):989–1004. 10.1016/j.immuni.2016.05.001 27192565 PMC4942846

[48] Liang B Workman C Lee J Chew C Dale BM Colonna L Regulatory T cells inhibit dendritic cells by lymphocyte activation gene-3 engagement of MHC class II. The J Immunol (2008) 180(9):5916–26. 10.4049/jimmunol.180.9.5916 18424711

[49] Maruhashi T Sugiura D Okazaki IM Shimizu K Maeda TK Ikubo J Binding of LAG-3 to stable peptide-MHC class II limits T cell function and suppresses autoimmunity and anti-cancer immunity. Immunity (2022) 55(5):912–24.e8. 10.1016/j.immuni.2022.03.013 35413245

[50] Wang J Sanmamed MF Datar I Su TT Ji L Sun J Fibrinogen-like protein 1 is a major immune inhibitory ligand of LAG-3. Cell (2019) 176(1-2):334–47.e12. 10.1016/j.cell.2018.11.010 30580966 PMC6365968

[51] Abdelhakim H Dunavin N Li M Braun M Elkhanany A Lin T LAG3 promotes acute myeloid leukemia-induced immune suppression. Blood (2018) 132(Suppl. 1):2414–4. 10.1182/blood-2018-99-117784

[52] Gautron AS Dominguez-Villar M de Marcken M Hafler DA. Enhanced suppressor function of TIM-3+ FoxP3+ regulatory T cells. Eur J Immunol (2014) 44(9):2703–11. 10.1002/eji.201344392 24838857 PMC4165702

[53] Dama P Tang M Fulton N Kline J Liu H. Gal9/Tim-3 expression level is higher in AML patients who fail chemotherapy. J Immunother Cancer (2019) 7(1):175. 10.1186/s40425-019-0611-3 31291985 PMC6621946

[54] Alqahtani M Aljuaimlani A Al-Tamimi J Alomar S Mansour L. TIM3 and CTLA4 immune checkpoint polymorphisms are associated with acute myeloid leukemia in Saudi Arabia. Hematology (2024) 29(1):2329024. 10.1080/16078454.2024.2329024 38536023

[55] Daver N Garcia-Manero G Basu S Boddu PC Alfayez M Cortes JE Efficacy, safety, and biomarkers of response to azacitidine and nivolumab in relapsed/refractory acute myeloid leukemia: a nonrandomized, open-label, phase II study. Cancer Discov (2019) 9(3):370–83. 10.1158/2159-8290.cd-18-0774 30409776 PMC6397669

[56] Gojo I Stuart R Webster J Blackford A Varela J Morrow J Multi-center phase 2 study of pembroluzimab (pembro) and azacitidine (AZA) in patients with relapsed/refractory acute myeloid leukemia (AML) and in newly diagnosed (≥65 Years) AML patients. Blood (2019) 134(Suppl. 1):832–2. 10.1182/blood-2019-127345

[57] Chien KS Kim K Nogueras-Gonzalez GM Borthakur G Naqvi K Daver NG Phase II study of azacitidine with pembrolizumab in patients with intermediate-1 or higher-risk myelodysplastic syndrome. Br J Haematol (2021) 195(3):378–87. 10.1111/bjh.17689 34340254

[58] Yang H Bueso-Ramos C DiNardo C Estecio MR Davanlou M Geng QR Expression of PD-L1, PD-L2, PD-1 and CTLA4 in myelodysplastic syndromes is enhanced by treatment with hypomethylating agents. Leukemia (2014) 28(6):1280–8. 10.1038/leu.2013.355 24270737 PMC4032802

[59] Zeidner JF Vincent BG Ivanova A Moore D McKinnon KP Wilkinson AD Phase II trial of pembrolizumab after high-dose cytarabine in relapsed/refractory acute myeloid leukemia. Blood Cancer Discov (2021) 2(6):616–29. 10.1158/2643-3230.bcd-21-0070 34778801 PMC8580622

[60] Daver N Garcia-Manero G Konopleva M Alfayez M Pemmaraju N Kadia T Azacitidine (aza) with nivolumab (nivo), and aza with nivo + ipilimumab (ipi) in relapsed/refractory acute myeloid leukemia: a non-randomized, prospective, phase 2 study. Blood (2019) 134(Suppl. 1):830–0. 10.1182/blood-2019-131494

[61] Oran B Garcia-Manero G Saliba R Alatrash G Jabbour E Popat U Post allogeneic stem cell transplant (SCT) cyclophosphamide improves progression free survival (PFS) in pts with AML/MDS treated with CTLA-4 or PD-1 blockade prior to SCT. Blood (2018) 132(Suppl. 1):483–3. 10.1182/blood-2018-99-115382

[62] Zheng H Mineishi S Claxton D Zhu J Zhao C Jia B A phase I clinical trial of avelumab in combination with decitabine as first line treatment of unfit patients with acute myeloid leukemia. Am J Hematol (2021) 96(2):E46–e50. 10.1002/ajh.26043 33146922 PMC7894154

[63] Gerds AT Scott BL Greenberg P Lin TL Pollyea DA Verma A Atezolizumab alone or in combination did not demonstrate a favorable risk-benefit profile in myelodysplastic syndrome. Blood Adv (2022) 6(4):1152–61. 10.1182/bloodadvances.2021005240 34932793 PMC8864663

[64] Zhong RK Loken M Lane TA Ball ED. CTLA-4 blockade by a human MAb enhances the capacity of AML-derived DC to induce T-cell responses against AML cells in an autologous culture system. Cytotherapy (2006) 8(1):3–12. 10.1080/14653240500499507 16627340

[65] Jones B Liu C Swiecki M Strake B Chi E Bansal-Pakala P. An agonist TIGIT mab suppresses regulatory T cell activity via an IL-2-mediated mechanism. The J Immunol (2019) 202(1_Suppl. e):57.9. 10.4049/jimmunol.202.supp.57.9

[66] Kong Y Zhu L Schell TD Zhang J Claxton DF Ehmann WC T-cell immunoglobulin and ITIM domain (TIGIT) associates with CD8+ T-cell exhaustion and poor clinical outcome in AML patients. Clin Cancer Res (2016) 22(12):3057–66. 10.1158/1078-0432.ccr-15-2626 26763253

[67] Yamamoto N Koyama T Sato J Yoshida T Sudo K Iwasa S Phase I study of the anti-TIGIT antibody tiragolumab in combination with atezolizumab in Japanese patients with advanced or metastatic solid tumors. Cancer Chemother Pharmacol (2024) 94(1):109–15. 10.1007/s00280-023-04627-3 38206370 PMC11258096

[68] Schwartz S Patel N Longmire T Jayaraman P Jiang X Lu H Characterization of sabatolimab, a novel immunotherapy with immuno-myeloid activity directed against TIM-3 receptor. Immunother Adv (2022) 2(1):ltac019. 10.1093/immadv/ltac019 36196369 PMC9525012

[69] Sordo-Bahamonde C Lorenzo-Herrero S González-Rodríguez AP Payer Á González-García E López-Soto A LAG-3 blockade with relatlimab (BMS-986016) restores anti-leukemic responses in chronic lymphocytic leukemia. Cancers (2021) 13(9):2112. 10.3390/cancers13092112 33925565 PMC8123840

[70] Han Y Dong Y Yang Q Xu W Jiang S Yu Z Acute myeloid leukemia cells express ICOS ligand to promote the expansion of regulatory T cells. Front Immunol (2018) 9:2227. 10.3389/fimmu.2018.02227 30319662 PMC6168677

[71] Pan S Cai Q Wei Y Tang H Zhang Y Zhou W Increased co-expression of ICOS and PD-1 predicts poor overall survival in patients with acute myeloid leukemia. Immunobiology (2024) 229(3):152804. 10.1016/j.imbio.2024.152804 38615511

[72] Wan Y Zhang C Xu Y Wang M Rao Q Xing H Hyperfunction of CD4 CD25 regulatory T cells in *de novo* acute myeloid leukemia. BMC cancer (2020) 20(1):472. 10.1186/s12885-020-06961-8 32456622 PMC7249438

[73] Wang R Feng W Wang H Wang L Yang X Yang F Blocking migration of regulatory T cells to leukemic hematopoietic microenvironment delays disease progression in mouse leukemia model. Cancer Lett (2020) 469:151–61. 10.1016/j.canlet.2019.10.032 31669202

[74] Modak RV de Oliveira Rebola KG McClatchy J Mohammadhosseini M Damnernsawad A Kurtz SE Targeting CCL2/CCR2 signaling overcomes MEK inhibitor resistance in acute myeloid leukemia. Clin Cancer Res (2024) 30(10):2245–59. 10.1158/1078-0432.ccr-23-2654 38451486 PMC11094423

[75] Zhu B Pan S Liu J Wang S Ni Y Xiao L HIF-1α forms regulatory loop with YAP to coordinate hypoxia-induced adriamycin resistance in acute myeloid leukemia cells. Cell Biol Int (2020) 44(2):456–66. 10.1002/cbin.11246 31617641

[76] Miska J Lee-Chang C Rashidi A Muroski ME Chang AL Lopez-Rosas A HIF-1α is a metabolic switch between glycolytic-driven migration and oxidative phosphorylation-driven immunosuppression of Tregs in glioblastoma. Cell Rep (2022) 39(10):110934. 10.1016/j.celrep.2022.110934 35675772

[77] Amin AH Sharifi LMA Kakhharov AJ Opulencia MJC Alsaikhan F Bokov DO Role of Acute Myeloid Leukemia (AML)-Derived exosomes in tumor progression and survival. Biomed and Pharmacother (2022) 150:113009. 10.1016/j.biopha.2022.113009 35486974

[78] Chen T Zhang G Kong L Xu S Wang Y Dong M. Leukemia-derived exosomes induced IL-8 production in bone marrow stromal cells to protect the leukemia cells against chemotherapy. Life Sci (2019) 221:187–95. 10.1016/j.lfs.2019.02.003 30716336

[79] Hong CS Sharma P Yerneni SS Simms P Jackson EK Whiteside TL Circulating exosomes carrying an immunosuppressive cargo interfere with cellular immunotherapy in acute myeloid leukemia. Scientific Rep (2017) 7(1):14684. 10.1038/s41598-017-14661-w PMC5666018 29089618

[80] Norsworthy KJ Ko CW Lee JE Liu J John CS Przepiorka D FDA approval summary: mylotarg for treatment of patients with relapsed or refractory CD33-positive acute myeloid leukemia. The Oncologist (2018) 23(9):1103–8. 10.1634/theoncologist.2017-0604 29650683 PMC6192608

[81] Jen EY Ko CW Lee JE Del Valle PL Aydanian A Jewell C FDA approval: gemtuzumab ozogamicin for the treatment of adults with newly diagnosed CD33-positive acute myeloid leukemia. Clin Cancer Res (2018) 24(14):3242–6. 10.1158/1078-0432.ccr-17-3179 29476018

[82] Wei A Fong C Montesinos P Calbacho M Gil J Perez De Oteyza J A phase 1 study of flotetuzumab, a CD123 x CD3 DART® protein, combined with MGA012, an anti-PD-1 antibody, in patients with relapsed or refractory acute myeloid leukemia. Blood (2019) 134(Suppl. 1):2662–2. 10.1182/blood-2019-125966

[83] Daver N Montesinos P DeAngelo D Wang E Todisco E Tarella C A phase I/II study of IMGN632, a novel CD123-targeting antibody-drug conjugate, in patients with relapsed/refractory acute myeloid leukemia, blastic plasmacytoid dendritic cell neoplasm, and other CD123-positive hematologic malignancies. J Clin Oncol (2020) 38(15_Suppl. l):Tps7563–tps63. 10.1200/jco.2020.38.15_suppl.tps7563

[84] Tahk S Vick B Hiller B Schmitt S Marcinek A Perini ED SIRPα-αCD123 fusion antibodies targeting CD123 in conjunction with CD47 blockade enhance the clearance of AML-initiating cells. J Hematol and Oncol (2021) 14(1):155. 10.1186/s13045-021-01163-6 34579739 PMC8477557

[85] Wilson NR Bover L Konopleva M Han L Neelapu S Pemmaraju N. CD303 (BDCA-2) - a potential novel target for therapy in hematologic malignancies. Leuk and Lymphoma (2022) 63(1):19–30. 10.1080/10428194.2021.1975192 34486917

[86] Xiao W Chan A Waarts MR Mishra T Liu Y Cai SF Plasmacytoid dendritic cell expansion defines a distinct subset of RUNX1-mutated acute myeloid leukemia. Blood (2021) 137(10):1377–91. 10.1182/blood.2020007897 32871587 PMC7955409

[87] Zhu L Wang P Zhang W Li Q Xiong J Li J Plasmacytoid dendritic cell infiltration in acute myeloid leukemia. Cancer Manag Res (2020) 12:11411–9. 10.2147/cmar.s260825 33192098 PMC7654521

[88] Ocadlikova D Casella S Pintao I Adinolfi E Sangaletti S De Marchi E Mechanisms of tolerance induction through T regulatory cells during chemotherapy-mediated immunogenic cell death in acute myeloid leukemia. Blood (2019) 134(Suppl. 1):2332–2. 10.1182/blood-2019-126478

[89] Parisi S Trabanelli S Ocadlikova D Paolini S Papayannidis C Ottaviani E Indoleamine 2,3-dioxygenase (Ido) is associated with high incidence of chemorefractory disease in acute myeloid leukemia (AML) patients. Blood (2012) 120(21):4787–7. 10.1182/blood.v120.21.4787.4787

[90] Sarangi P. Role of indoleamine 2, 3-dioxygenase 1 in immunosuppression of breast cancer. Cancer Pathogenesis Ther (2024) 2(4):246–55. 10.1016/j.cpt.2023.11.001 PMC11447360 39371092

[91] Chamuleau ME van de Loosdrecht AA Hess CJ Janssen JJ Zevenbergen A Delwel R High INDO (indoleamine 2,3-dioxygenase) mRNA level in blasts of acute myeloid leukemic patients predicts poor clinical outcome. Haematologica (2008) 93(12):1894–8. 10.3324/haematol.13113 19050070

[92] Schmidt A Oberle N Krammer PH. Molecular mechanisms of treg-mediated T cell suppression. Front Immunol (2012) 3:51. 10.3389/fimmu.2012.00051 22566933 PMC3341960

[93] Onishi Y Fehervari Z Yamaguchi T Sakaguchi S. Foxp3+ natural regulatory T cells preferentially form aggregates on dendritic cells *in vitro* and actively inhibit their maturation. Proc Natl Acad Sci (2008) 105(29):10113–8. 10.1073/pnas.0711106105 18635688 PMC2481354

[94] Wilson KR Villadangos JA Mintern JD. Dendritic cell Flt3 - regulation, roles and repercussions for immunotherapy. Immunol and Cell Biol (2021) 99(9):962–71. 10.1111/imcb.12484 34097779

[95] Yamamoto Y Kiyoi H Nakano Y Suzuki R Kodera Y Miyawaki S Activating mutation of D835 within the activation loop of FLT3 in human hematologic malignancies. Blood (2001) 97(8):2434–9. 10.1182/blood.v97.8.2434 11290608

[96] Vacchelli E Vitale I Eggermont A Fridman WH Fučíková J Cremer I Trial watch: dendritic cell-based interventions for cancer therapy. Oncoimmunology (2013) 2(10):e25771. 10.4161/onci.25771 24286020 PMC3841205

[97] Zhang L Xu Y Shen J He F Zhang D Chen Z Feasibility study of DCs/CIKs combined with thoracic radiotherapy for patients with locally advanced or metastatic non-small-cell lung cancer. Radiat Oncol (London, England) (2016) 11:60. 10.1186/s13014-016-0635-5 PMC4839093 27097970

[98] Yanagisawa R Koizumi T Koya T Sano K Koido S Nagai K WT1-pulsed dendritic cell vaccine combined with chemotherapy for resected pancreatic cancer in a phase I study. Anticancer Res (2018) 38(4):2217–25. 10.21873/anticanres.12464 29599342

[99] Pepeldjiyska E Li L Gao J Seidel CL Blasi C Özkaya E Leukemia derived dendritic cell (DCleu) mediated immune response goes along with reduced (leukemia-specific) regulatory T-cells. Immunobiology (2022) 227(4):152237. 10.1016/j.imbio.2022.152237 35749805

[100] Huntington ND Cursons J Rautela J. The cancer-natural killer cell immunity cycle. Nat Rev Cancer (2020) 20(8):437–54. 10.1038/s41568-020-0272-z 32581320

[101] Yang L Feng Y Wang S Jiang S Tao L Li J Siglec-7 is an indicator of natural killer cell function in acute myeloid leukemia. Int immunopharmacology (2021) 99:107965. 10.1016/j.intimp.2021.107965 34273636

[102] Djaoud Z Parham P. HLAs, TCRs, and KIRs, a triumvirate of human cell-mediated immunity. Annu Rev Biochem (2020) 89:717–39. 10.1146/annurev-biochem-011520-102754 32569519

[103] Schetelig J Baldauf H Heidenreich F Massalski C Frank S Sauter J External validation of models for KIR2DS1/KIR3DL1-informed selection of hematopoietic cell donors fails. Blood (2020) 135(16):1386–95. 10.1182/blood.2019002887 31932846 PMC7162689

[104] Cianga VA Campos Catafal L Cianga P Pavel Tanasa M Cherry M Collet P Natural killer cell subpopulations and inhibitory receptor dynamics in myelodysplastic syndromes and acute myeloid leukemia. Front Immunol (2021) 12:665541. 10.3389/fimmu.2021.665541 33986753 PMC8112610

[105] Dai YJ He SY Hu F Li XP Zhang JM Chen SL Bone marrow infiltrated natural killer cells predicted the anti-leukemia activity of MCL1 or BCL2 inhibitors in acute myeloid leukemia. Mol Cancer (2021) 20(1):8. 10.1186/s12943-020-01302-6 33402171 PMC7784307

[106] Liu L Chen X Jin HM Zhao SS Zhu Y Qian SX The expression and function of NK cells in patients with acute myeloid leukemia. Zhongguo shi yan xue ye xue za zhi (2022) 30(1):49–55. 10.19746/j.cnki.issn.1009-2137.2022.01.009 35123603

[107] Russick J Torset C Hemery E Cremer I. NK cells in the tumor microenvironment: prognostic and theranostic impact. Recent advances and trends. Semin Immunol (2020) 48:101407. 10.1016/j.smim.2020.101407 32900565

[108] Kaweme NM Zhou F. Optimizing NK cell-based immunotherapy in myeloid leukemia: abrogating an immunosuppressive microenvironment. Front Immunol (2021) 12:683381. 10.3389/fimmu.2021.683381 34220833 PMC8247591

[109] Zhang T Fang Q Liu P Wang P Feng C Wang J. Heme oxygenase 1 overexpression induces immune evasion of acute myeloid leukemia against natural killer cells by inhibiting CD48. J translational Med (2022) 20(1):394. 10.1186/s12967-022-03589-z PMC9441067 36058936

[110] Chajuwan T Kansuwan P Kobbuaklee S Chanswangphuwana C. Characteristics and clinical correlation of TIM-3 and PD-1/PD-L1 expressions in leukemic cells and tumor microenvironment in newly diagnosed acute myeloid leukemia. Leuk and Lymphoma (2022) 63(2):450–6. 10.1080/10428194.2021.1984454 34585994

[111] Bou-Tayeh B Laletin V Salem N Just-Landi S Fares J Leblanc R Chronic IL-15 stimulation and impaired mTOR signaling and metabolism in natural killer cells during acute myeloid leukemia. Front Immunol (2021) 12:730970. 10.3389/fimmu.2021.730970 34975835 PMC8718679

[112] Baragaño Raneros A Martín-Palanco V Fernandez AF Rodriguez RM Fraga MF Lopez-Larrea C Methylation of NKG2D ligands contributes to immune system evasion in acute myeloid leukemia. Genes Immun (2015) 16(1):71–82. 10.1038/gene.2014.58 25393931

[113] Belizário JE Neyra JM Setúbal Destro Rodrigues MF. When and how NK cell-induced programmed cell death benefits immunological protection against intracellular pathogen infection. Innate Immun (2018) 24(8):452–65. 10.1177/1753425918800200 30236030 PMC6830868

[114] Zhang Q Bi J Zheng X Chen Y Wang H Wu W Blockade of the checkpoint receptor TIGIT prevents NK cell exhaustion and elicits potent anti-tumor immunity. Nat Immunol (2018) 19(7):723–32. 10.1038/s41590-018-0132-0 29915296

[115] Wang M Bu J Zhou M Sido J Lin Y Liu G CD8+T cells expressing both PD-1 and TIGIT but not CD226 are dysfunctional in acute myeloid leukemia (AML) patients. Clin Immunol (2018) 190:64–73. 10.1016/j.clim.2017.08.021 28893624

[116] Coles SJ Wang EC Man S Hills RK Burnett AK Tonks A CD200 expression suppresses natural killer cell function and directly inhibits patient anti-tumor response in acute myeloid leukemia. Leukemia (2011) 25(5):792–9. 10.1038/leu.2011.1 21274000 PMC3093357

[117] Tonks A Hills R White P Rosie B Mills KI Burnett AK CD200 as a prognostic factor in acute myeloid leukaemia. Leukemia (2007) 21(3):566–8. 10.1038/sj.leu.2404559 17252007

[118] Xiao Y Chen J Wang J Guan W Wang M Zhang L Acute myeloid leukemia epigenetic immune escape from nature killer cells by ICAM-1. Front Oncol (2021) 11:751834. 10.3389/fonc.2021.751834 34722306 PMC8548470

[119] Wang Z Xiao Y Guan W Wang M Chen J Zhang L Acute myeloid leukemia immune escape by epigenetic CD48 silencing. Clin Sci (2020) 134(2):261–71. 10.1042/cs20191170 31922199

[120] Ewen EM Pahl JHW Miller M Watzl C Cerwenka A. KIR downregulation by IL-12/15/18 unleashes human NK cells from KIR/HLA-I inhibition and enhances killing of tumor cells. Eur J Immunol (2018) 48(2):355–65. 10.1002/eji.201747128 29105756

[121] Satwani P van de Ven C Ayello J Cairo D Simpson LL Baxi L Interleukin (IL)-15 in combination with IL-2, fms-like tyrosine kinase-3 ligand and anti-CD3 significantly enhances umbilical cord blood natural killer (NK) cell and NK-cell subset expansion and NK function. Cytotherapy (2011) 13(6):730–8. 10.3109/14653249.2011.563292 21413839

[122] Wang QS Wang Y Lv HY Han QW Fan H Guo B Treatment of CD33-directed chimeric antigen receptor-modified T cells in one patient with relapsed and refractory acute myeloid leukemia. Mol Ther (2015) 23(1):184–91. 10.1038/mt.2014.164 25174587 PMC4426796

[123] Yao S Jianlin C Yarong L Botao L Qinghan W Hongliang F Donor-derived cd123-targeted CAR T cell serves as a RIC regimen for haploidentical transplantation in a patient with FUS-ERG+ AML. Front Oncol (2019) 9:1358. 10.3389/fonc.2019.01358 31850234 PMC6901822

[124] You L Han Q Zhu L Zhu Y Bao C Yang C Decitabine-mediated epigenetic reprograming enhances anti-leukemia efficacy of cd123-targeted chimeric antigen receptor T-cells. Front Immunol (2020) 11:1787. 10.3389/fimmu.2020.01787 32973749 PMC7461863

[125] Raulet DH Gasser S Gowen BG Deng W Jung H. Regulation of ligands for the NKG2D activating receptor. Annu Rev Immunol (2013) 31:413–41. 10.1146/annurev-immunol-032712-095951 23298206 PMC4244079

[126] Sallman D Al-Homsi A Davila M Kerre T Moors I Poire X Results from the phase I clinical studies evaluating cyad-01, a first-generation NKG2D CAR T-cell product in relapsed or refractory acute myeloid leukemia and myelodysplastic syndrome patients. Blood (2020) 136(Suppl. 1):40–1. 10.1182/blood-2020-139609

[127] Xie G Dong H Liang Y Ham JD Rizwan R Chen J. CAR-NK cells: a promising cellular immunotherapy for cancer. EBioMedicine (2020) 59:102975. 10.1016/j.ebiom.2020.102975 32853984 PMC7452675

[128] Marofi F Saleh MM Rahman HS Suksatan W Al-Gazally ME Abdelbasset WK CAR-engineered NK cells; a promising therapeutic option for treatment of hematological malignancies. Stem Cel Res and Ther (2021) 12(1):374. 10.1186/s13287-021-02462-y PMC8252313 34215336

[129] Lu H Zhao X Li Z Hu Y Wang H. From CAR-T cells to CAR-NK cells: a developing immunotherapy method for hematological malignancies. Front Oncol (2021) 11:720501. 10.3389/fonc.2021.720501 34422667 PMC8377427

[130] Zhou Q Bucher C Munger ME Highfill SL Tolar J Munn DH Depletion of endogenous tumor-associated regulatory T cells improves the efficacy of adoptive cytotoxic T-cell immunotherapy in murine acute myeloid leukemia. Blood (2009) 114(18):3793–802. 10.1182/blood-2009-03-208181 19724059 PMC2773484

[131] Greiner J Götz M Hofmann S Schrezenmeier H Wiesneth M Bullinger L Specific T-cell immune responses against colony-forming cells including leukemic progenitor cells of AML patients were increased by immune checkpoint inhibition. Cancer Immunol Immunother (2020) 69(4):629–40. 10.1007/s00262-020-02490-2 32020256 PMC11027801

[132] Rakova J Truxova I Holicek P Salek C Hensler M Kasikova L TIM-3 levels correlate with enhanced NK cell cytotoxicity and improved clinical outcome in AML patients. Oncoimmunology (2021) 10(1):1889822. 10.1080/2162402x.2021.1889822 33758676 PMC7946028

[133] Abolhalaj M Sincic V Lilljebjörn H Sandén C Aab A Hägerbrand K Transcriptional profiling demonstrates altered characteristics of CD8^+^ cytotoxic T‐cells and regulatory T‐cells in *TP53*mutated acute myeloid leukemia. Cancer Med (2022) 11(15):3023–32. 10.1002/cam4.4661 35297213 PMC9359873

[134] Song X Peng Y Wang X Chen Q Lan X Shi F. The stimulator of interferon genes (STING) agonists for treating acute myeloid leukemia (AML): current knowledge and future outlook. Clin Translational Oncol (2022) 25:1545–53. 10.1007/s12094-022-03065-6 36587109

[135] Xue L Hu Y Wang J Liu X Wang X. T cells targeting multiple tumor-associated antigens as a postremission treatment to prevent or delay relapse in acute myeloid leukemia. Cancer Manag Res (2019) 11:6467–76. 10.2147/cmar.s205296 31406473 PMC6642655

[136] Zhang M Sukhumalchandra P Philips A Qiao N Kerros C Molldrem JJ Pembrolizumab enhances the anti-leukemia activity of antigen specific cytotoxic T lymphocytes. Blood (2018) 132(Suppl. 1):4542–2. 10.1182/blood-2018-99-114864

[137] Gibson A Zhang M Sukhumalchandra P Philips A Qiao N Perakis A Pembrolizumab in combination with antigen-specific cytotoxic T lymphocytes enhances killing of acute myeloid leukemia. Blood (2021) 138(Suppl. 1):2775–5. 10.1182/blood-2021-149318

[138] Chapuis AG Egan DN Bar M Schmitt TM McAfee MS Paulson KG T cell receptor gene therapy targeting WT1 prevents acute myeloid leukemia relapse post-transplant. Nat Med (2019) 25(7):1064–72. 10.1038/s41591-019-0472-9 31235963 PMC6982533

[139] Morris E Tendeiro-Rego R Richardson R Fox T Sillito F Holler A A phase I study evaluating the safety and persistence of allorestricted WT1-TCR gene modified autologous T cells in patients with high-risk myeloid malignancies unsuitable for allogeneic stem cell transplantation. Blood (2019) 134(Suppl. 1):1367–7. 10.1182/blood-2019-128044 31698429

[140] Maniati E Soper R Hagemann T. Up for Mischief? IL-17/Th17 in the tumour microenvironment. Oncogene (2010) 29(42):5653–62. 10.1038/onc.2010.367 20729908 PMC2962667

[141] Xu ZJ Gu Y Wang CZ Jin Y Wen XM Ma JC The M2 macrophage marker CD206: a novel prognostic indicator for acute myeloid leukemia. Oncoimmunology (2020) 9(1):1683347. 10.1080/2162402x.2019.1683347 32002295 PMC6959428

[142] Su X Ye J Hsueh EC Zhang Y Hoft DF Peng G. Tumor microenvironments direct the recruitment and expansion of human Th17 cells. The J Immunol (2010) 184(3):1630–41. 10.4049/jimmunol.0902813 20026736

[143] Zhang GL Pan M Wang YZ Huang JX Gu GS Wang Y Regulation effect of myeloid leukemia No.1 Chinese herb medicine prescription combined with chemotherapy on Th17 cells in bone marrow of patients with acute myeloid leukemia. Zhongguo shi yan xue ye xue za zhi (2021) 29(2):328–32. 10.19746/j.cnki.issn.1009-2137.2021.02.004 33812395

[144] Ren Z Huang X Lv Q Lei Y Shi H Wang F High expression of B4GALT1 is associated with poor prognosis in acute myeloid leukemia. Front Genet (2022) 13:882004. 10.3389/fgene.2022.882004 36568388 PMC9780537

[145] Wang M Zhang C Tian T Zhang T Wang R Han F Increased regulatory T cells in peripheral blood of acute myeloid leukemia patients rely on tumor necrosis factor (TNF)-α-TNF receptor-2 pathway. Front Immunol (2018) 9:1274. 10.3389/fimmu.2018.01274 29922294 PMC5996048

[146] Han Y Ye A Bi L Wu J Yu K Zhang S. Th17 cells and interleukin-17 increase with poor prognosis in patients with acute myeloid leukemia. Cancer Sci (2014) 105(8):933–42. 10.1111/cas.12459 24890519 PMC4317867

[147] Dhakal D Kunwar L. Clinical expression of th17 related cytokinesin acute myeloid leukemia patients: a prospective descriptive study. Int J Adv Res (2022) 10(03):34–9. 10.21474/ijar01/14359

[148] Yang X Zheng H Dai J Li C Zhang R Wang X Epigenetic therapy targets Th1/Th17 polarization to reversing immune evasion and treating leukemia relapse post allogeneic stem cell transplantation in non-APL AML patients. Blood (2018) 132(Suppl. 1):3424–4. 10.1182/blood-2018-99-111671

[149] Wang X Huang R Wu W Xiong J Wen Q Zeng Y Amplifying STING activation by bioinspired nanomedicine for targeted chemo- and immunotherapy of acute myeloid leukemia. Acta Biomater (2023) 157:381–94. 10.1016/j.actbio.2022.11.007 36375786

[150] Bassani B Simonetti G Cancila V Fiorino A Ciciarello M Piva A ZEB1 shapes AML immunological niches, suppressing CD8 T cell activity while fostering Th17 cell expansion. Cell Rep (2024) 43(2):113794. 10.1016/j.celrep.2024.113794 38363677

[151] Swatler J Turos-Korgul L Kozlowska E Piwocka K. Immunosuppressive cell subsets and factors in myeloid leukemias. Cancers (2021) 13(6):1203. 10.3390/cancers13061203 33801964 PMC7998753

[152] Kapor S Santibanez JF. Myeloid-derived suppressor cells and mesenchymal stem/stromal cells in myeloid malignancies. J Clin Med (2021) 10(13):2788. 10.3390/jcm10132788 34202907 PMC8268878

[153] Rodriguez PC Hernandez CP Quiceno D Dubinett SM Zabaleta J Ochoa JB Arginase I in myeloid suppressor cells is induced by COX-2 in lung carcinoma. The J Exp Med (2005) 202(7):931–9. 10.1084/jem.20050715 16186186 PMC2213169

[154] Corzo CA Cotter MJ Cheng P Cheng F Kusmartsev S Sotomayor E Mechanism regulating reactive oxygen species in tumor-induced myeloid-derived suppressor cells. The J Immunol (2009) 182(9):5693–701. 10.4049/jimmunol.0900092 19380816 PMC2833019

[155] Yang L Huang J Ren X Gorska AE Chytil A Aakre M Abrogation of TGFβ signaling in mammary carcinomas recruits gr-1+CD11b+ myeloid cells that promote metastasis. Cancer cell (2008) 13(1):23–35. 10.1016/j.ccr.2007.12.004 18167337 PMC2245859

[156] Giallongo C Parrinello N Brundo MV Raccuia SA Di Rosa M La Cava P Myeloid derived suppressor cells in chronic myeloid leukemia. Front Oncol (2015) 5:107. 10.3389/fonc.2015.00107 26029664 PMC4432672

[157] Braun LM Zeiser R. Immunotherapy in myeloproliferative diseases. Cells (2020) 9(6):1559. 10.3390/cells9061559 32604862 PMC7349594

[158] Hyun SY Na EJ Jang JE Chung H Kim SJ Kim JS Immunosuppressive role of CD11b+ CD33+ HLA-DR- myeloid-derived suppressor cells-like blast subpopulation in acute myeloid leukemia. Cancer Med (2020) 9(19):7007–17. 10.1002/cam4.3360 32780544 PMC7541151

[159] Folgiero V Goffredo BM Filippini P Masetti R Bonanno G Caruso R Indoleamine 2,3-dioxygenase 1 (Ido1) activity in leukemia blasts correlates with poor outcome in childhood acute myeloid leukemia. Oncotarget (2014) 5(8):2052–64. 10.18632/oncotarget.1504 24903009 PMC4039144

[160] Mussai F De Santo C Abu-Dayyeh I Booth S Quek L McEwen-Smith RM Acute myeloid leukemia creates an arginase-dependent immunosuppressive microenvironment. Blood (2013) 122(5):749–58. 10.1182/blood-2013-01-480129 23733335 PMC3731930

[161] Haist M Stege H Grabbe S Bros M. The functional crosstalk between myeloid-derived suppressor cells and regulatory T cells within the immunosuppressive tumor microenvironment. Cancers (2021) 13(2):210. 10.3390/cancers13020210 33430105 PMC7827203

[162] Ren X Tao Q Wang H Zhang Q Zhou M Liu L Monocytic myeloid-derived suppressor cells but not monocytes predict poor prognosis of acute myeloid leukemia. Turkish J Hematol (2022) 39(4):230–6. 10.4274/tjh.galenos.2022.2022.0137 PMC9727724 35965420

[163] Tohumeken S Baur R Böttcher M Stoll A Loschinski R Panagiotidis K Palmitoylated proteins on AML-derived extracellular vesicles promote myeloid-derived suppressor cell differentiation via TLR2/akt/mTOR signaling. Cancer Res (2020) 80(17):3663–76. 10.1158/0008-5472.can-20-0024 32605996

[164] Li X Li Y Yu Q Xu L Fu S Wei C mTOR signaling regulates the development and therapeutic efficacy of PMN-MDSCs in acute GVHD. Front Cel Dev Biol (2021) 9:741911. 10.3389/fcell.2021.741911 PMC8733691 35004668

[165] Bai H Peng Y Li Y Duan J Fu W Liang X Cytarabine-induced TNFα promotes the expansion and suppressive functions of myeloid-derived suppressor cells in acute myeloid leukaemia. Scand J Immunol (2022) 95(6):e13158. 10.1111/sji.13158 35285047

[166] Pyzer A Stroopinsky D Rajabi H Washington A Cole L Jain S Acute myeloid leukemia cells export c-myc in extracellular vesicles driving a proliferation of immune-suppressive myeloid-derived suppressor cells. Blood (2016) 128(22):703–3. 10.1182/blood.v128.22.703.703

[167] Mehta R Chen X Antony J Szabolcs P. Myeloid derived suppressor cells (MDSC)-like acute myeloid leukemia (AML) cells are associated with resistance to cytotoxic effects of autologous (auto) T-lymphocytes (CTLs). Biol Blood Marrow Transplant (2015) 21(2):S191–S192. 10.1016/j.bbmt.2014.11.290

[168] Notarantonio AB Bertrand A Piucco R Fievet G Sartelet H Boulangé L Highly immunosuppressive myeloid cells correlate with early relapse after allogeneic stem cell transplantation. Exp Hematol and Oncol (2024) 13(1):50. 10.1186/s40164-024-00516-4 PMC11088072 38734654

[169] Wang L Jia B Claxton DF Ehmann WC Rybka WB Mineishi S VISTA is highly expressed on MDSCs and mediates an inhibition of T cell response in patients with AML. Oncoimmunology (2018) 7(9):e1469594. 10.1080/2162402x.2018.1469594 30228937 PMC6140587

[170] Jochems C Kwilas A Kim Y Brechbiel M Hodge J Newton R The Ido inhibitor INCB024360 to enhance dendritic cell immunogenicity and anti-tumor immunity *in vitro* . J Clin Oncol (2015) 33(15_Suppl. l):e14012–e12. 10.1200/jco.2015.33.15_suppl.e14012

[171] Komrokji RS Wei S Mailloux AW Zhang L Padron E Sallman D A phase II study to determine the safety and efficacy of the oral inhibitor of indoleamine 2,3-dioxygenase (Ido) enzyme INCB024360 in patients with myelodysplastic syndromes. Clin Lymphoma Myeloma Leuk (2019) 19(3):157–61. 10.1016/j.clml.2018.12.005 30713125

[172] Ogasawara M Ota S. Dendritic cell vaccination in acute leukemia patients induces reduction of myeloid-derived suppressor cells: immunological analysis of a pilot study. Blood (2014) 124(21):1113–3. 10.1182/blood.v124.21.1113.1113

[173] Miner S Ito S Tanimoto K Hensel N Chinian F Keyvanfar K Myeloid leukemias directly suppress T cell proliferation through STAT3 and arginase pathways. Blood (2013) 122(21):3885–5. 10.1182/blood.v122.21.3885.3885

